# Decoding atherosclerosis through lactylation: multi-omics integration with experimental validation

**DOI:** 10.3389/fcell.2026.1742425

**Published:** 2026-05-08

**Authors:** Yirong Ma, Qiming Li, Muge Wang, Qiang Wan, Junyu Lai, Jianguang Wu, Shuguang Wu

**Affiliations:** 1 Neurology Department, Jiangxi Province Hospital of Integrated Chinese and Western Medicine, Nanchang, China; 2 Department of Postgraduate, Jiangxi University of Chinese Medicine, Nanchang, China; 3 Cardiology Department, Affiliated Hospital of Jiangxi University of Chinese Medicine, Nanchang, China

**Keywords:** atherosclerosis, lactylation, machine learning, molecular dynamics, proteomics

## Abstract

**Background:**

Atherosclerosis (AS) remains a major cause of cardiovascular morbidity and mortality. Lactylation—a recently described post-translational modification linking cellular metabolism to gene regulation—has been implicated in vascular inflammation, yet its roles in AS are not fully defined.

**Method:**

We modelled AS by exposing HUVECs to oxidised LDL and performed data-independent proteomics. Public AS transcriptomes were integrated and batch-corrected; lactylation-related genes (LRGs) were profiled; consensus clustering and WGCNA defined subtypes/modules. Candidate biomarkers were prioritised by intersecting proteomic, differential and network features, then evaluated using an ensemble of 12 machine-learning algorithms with cross-validation and external validation. Immune infiltration (CIBERSORT) and single-cell data characterised immune contexts. Connectivity Map, molecular docking and molecular dynamics (MD) explored therapeutics. Core genes were validated by RT-qPCR, Western blotting and immunofluorescence in Apoe^−/−^ mice.

**Results:**

Proteomics identified 472 differentially expressed proteins; GEO analyses yielded 2,544 DEGs and WGCNA 2,059 module genes, converging on 25 candidates. The top ensemble (LASSO + GBM) achieved a mean AUC 0.979 across training and external sets, nominating UAP1, NRP1, QPRT and NDST1 as hub genes. These genes associated with immune-cell infiltration and showed prominent single-cell expression in macrophages and smooth muscle cells. RT-qPCR *in vivo* showed NRP1/NDST1/QPRT upregulated and UAP1 downregulated versus controls (all P < 0.05); WB/IF confirmed higher NRP1 and lower UAP1 protein abundance. CMap analysis suggested several candidate compounds. Among them, rivaroxaban was prioritised for further *in silico* evaluation because NRP1 emerged as a validated hub gene, and docking and molecular dynamics simulations supported a stable predicted interaction between rivaroxaban and NRP1.

**Conclusion:**

UAP1, NRP1, QPRT and NDST1 represent lactylation-linked biomarkers of AS with diagnostic potential and plausible mechanistic relevance within immune-vascular pathways. Computational screening further prioritised a putative NRP1–rivaroxaban interaction as a hypothesis-generating lead for future experimental validation.

## 1 Introduction

Atherosclerosis (AS) is a chronic vascular disorder marked by lipid deposits, inflammatory reactions, and fibrosis within the arterial intima, which significantly contribute to the pathology of cardiovascular diseases (Bäck et al., 2019). Global epidemiological studies show that nearly 17 million deaths annually are attributable to cardiovascular diseases and strokes stemming from AS, comprising approximately 31% of global mortality (Benjamin et al., 2019). AS pathogenesis is multifaceted, involving endothelial dysfunction, lipid accumulation, migration and proliferation of smooth muscle cells, and immune responses. In initial stages, low-density lipoprotein (LDL) accumulates under the arterial endothelium, impairing endothelial cells and fostering inflammatory cell infiltration, leading to foam cell formation. As lesions advance, atherosclerotic plaques not only narrow the vessel lumen but can also precipitate plaque rupture, potentially causing acute coronary syndrome (Jebari-Benslaiman et al., 2022; Zhang et al., 2023). Moreover, recent research underscores the pivotal roles of microRNAs, inflammatory mediators, and genomic variations in AS development, highlighting the synergy between genetic predisposition and environmental factors (Letonja and Petrovič, 2024). While treatment modalities such as statins, antihypertensive medications, and antiplatelet therapies have been effective in reducing cardiovascular event risks associated with AS (Wang et al., 2018), the efficacy of personalized treatments and early interventions remains to be further investigated to pioneer advancements in managing disease progression and improving outcomes. Consequently, due to the intricate mechanisms and multiple risk factors associated with AS, it is crucial to develop more precise interventions. Notably, identifying key genetic markers of AS could offer vital insights for the early detection and efficient management of the disease.

Lactate, traditionally viewed as merely a metabolic waste product, is not only an intermediate of glycolysis but also plays pivotal roles in various physiological and pathological processes. Recent studies have illuminated lactate’s function in intracellular signaling and epigenetic regulation, particularly its role in histone lactylation, which challenges the conventional understanding of lactate (Hu et al., 2024). Research demonstrates that lactate directly influences chromatin structure and gene expression through its lactylation, a modification analogous to acetylation in its regulatory impact on gene expression (Galle et al., 2022). Notably, the discovery of lactylation, especially in the adaptive survival strategies of tumor cells, positions lactate as a signaling molecule that alters cell fate by modifying protein functions and locations (Gong et al., 2024; Wang G. et al., 2024). Moreover, the role of lactate in immune regulation is evidenced by its significant impact on T cell migration and function in high lactate environments, which is crucial in chronic inflammatory and autoimmune diseases (Haas et al., 2015). Further studies indicate that lactate and its lactylation modifications are instrumental in the pathophysiology of AS. Lactate accumulation in vascular endothelial and smooth muscle cells, often a direct response to metabolic stressors such as hypoxia or inflammation, may regulate the expression of key genes involved in inflammation and vascular remodeling through its lactylation modifications (Fan et al., 2024). This modification may facilitate the transition of vascular smooth muscle cells from contractile to synthetic phenotypes, exacerbating inflammatory responses and vascular structural changes, thereby playing a critical role in plaque formation and progression (Lambert and Jørgensen, 2021; Cao et al., 2022). Elucidating these mechanisms not only provides new insights into the complex pathobiology of AS but also suggests potential new targets for preventive and therapeutic strategies. Consequently, identifying LRGs in AS patients may offer novel approaches for the diagnosis and treatment of this disease.

**FIGURE 1:**
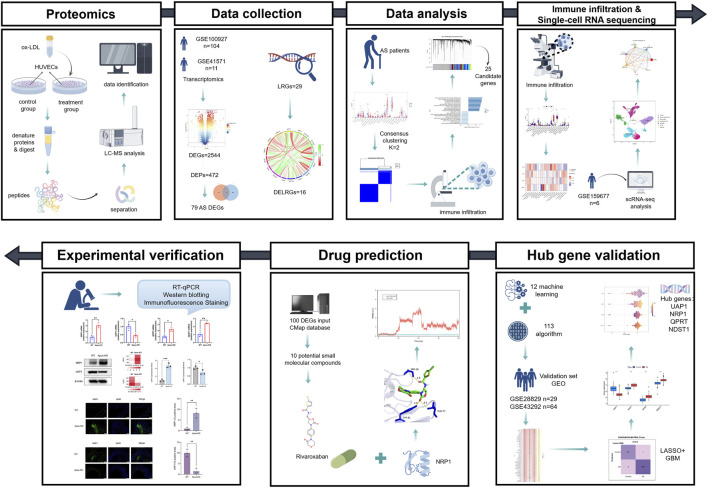
Schematic overview of the study design and analytical workflow. The workflow includes data collection and preprocessing, identification of lactylation-related genes and molecular subtypes, differential expression and functional enrichment analyses, machine learning-based hub gene screening, immune infiltration and single-cell analyses, drug–target interaction analysis, and experimental validation. By figdraw.

In this study, we performed a comprehensive analysis utilizing multi-omics data and machine learning algorithms, and experimentally validated the expression and localization of hub genes associated with lactylation in AS patients. Using the CIBERSORT algorithm and single-cell sequencing data, we further investigated immune cell infiltration in AS patients. Additionally, through CMap analysis and molecular dynamics simulations, we uncovered the potential therapeutic mechanisms of Rivaroxaban in AS. The research workflow is presented in Figure 1. This study provides valuable preliminary scientific evidence to support the development of precise diagnostic and targeted therapeutic strategies for AS, with the goal of informing the application of personalized medicine in cardiovascular disease management.

## 2 Materials and methods

### 2.1 Cell cultivation and treatment

Human umbilical vein endothelial cells (HUVEC, RRID: CVCL_2959) were used in this study and were purchased from Wuhan PunoSa Biotechnology Co., Ltd. (Cat. No. CP-H082, March 2024). The cells are derived from the endothelium of the human umbilical vein. Authentication was performed by the supplier using STR profiling, showing a >95% match with the reference profile of HUVEC. No misidentification or contamination has been reported in public databases. Routine PCR-based screening for *mycoplasma* was carried out and found to be consistently negative. After thawing, the cells were cultured in ECM endothelial cell medium supplemented with 10% fetal bovine serum and 1% penicillin/streptomycin at 37 °C in a humidified atmosphere containing 5% CO_2_. Cells were passaged to the third generation and used during the logarithmic growth phase for subsequent experiments. Two experimental groups were established: a blank control group, in which cells received an equal volume of culture medium only, and a model group, in which cells were treated with 150 μM commercially obtained oxidized low-density lipoprotein (ox-LDL; Yiyuan Biotechnology, Cat. No. YB-002) for 24 h to establish the *in vitro* AS model. After treatment, the cells were harvested for proteomic analysis.

### 2.2 Protein extraction and pre-processing

Following cell treatment, the cells were washed three times with PBS solution, and then 80 μL of lysis buffer containing 10% C12Im-Cl ionic liquid and 0.1% protease inhibitor was added. The cells underwent sonication for 100 s using a non-contact ultrasonic homogenizer, applying a cycle of 20 s of sonication followed by 30 s of cooling, repeated five times, to extract total cellular proteins. Subsequently, DTT was introduced to the protein extract to achieve a final concentration of 100 mM, and the mixture was denatured at 95 °C for 5 min. The denatured sample was then transferred to a 10 kDa molecular weight cut-off membrane and centrifuged at 16,000 g for 30 min to eliminate small molecular impurities. The sample was subsequently washed with 50 mM phosphate buffer and treated with 50 mM iodoacetamide (IAA) for alkylation, incubating in the dark for 30 min before being centrifuged at 16,000 g to remove excess unreacted IAA. After four washes with 50 mM phosphate buffer, trypsin was added at an enzyme-to-protein mass ratio of 1:30, and the sample was digested overnight at 37 °C. The following day, the resulting peptide solution was collected via centrifugation at 16,000 g and stored at −20 °C for subsequent mass spectrometric analysis.

### 2.3 Proteomics analysis and data processing

DIA raw files were processed in Spectronaut (v17, Biognosys) using the library-free DirectDIA workflow. A spectral library was generated on-the-fly from the DIA data with Pulsar. Carbamidomethyl (C) was set as a fixed modification, and oxidation (M) and protein N-terminal acetylation were set as variable modifications. False discovery rate (FDR) control was set to 1% at the precursor, peptide and protein levels (q-value). Cross-run normalisation used Spectronaut’s Local Normalisation algorithm with dynamic iRT-based retention-time calibration; interference correction was enabled. Protein quantities were summarised from peptide-level MS2 peak areas using Spectronaut’s default protein inference and summarisation settings. Unless otherwise stated, all parameters followed the vendor’s recommendations.

### 2.4 Data collection

We retrieved four atherosclerosis-related microarray datasets from the GEO database (GSE100927, GSE41571, GSE28829, and GSE43292) according to the following criteria: expression profiling by microarray, Homo sapiens as the study subject, arterial tissue samples, and inclusion of both AS and control samples with a minimum sample size of five per group. For transcriptomic discovery analyses, only GSE100927 and GSE41571 were integrated, whereas GSE28829 and GSE43292 were reserved as independent external validation cohorts (Table 1). For each discovery dataset, the normalized gene-expression matrix was used as input. Duplicate gene symbols were collapsed using the avereps function in the limma package, and only the intersecting genes shared across datasets were retained for integration. The expression matrices were then merged by common genes, and the dataset source was encoded as the batch variable. Inter-dataset batch effects were corrected using the ComBat function in the sva package with empirical Bayes adjustment (par.prior = TRUE) (Chakraborty et al., 2012). The batch-corrected merged matrix was subsequently used for downstream differential expression analysis, consensus clustering, WGCNA, and machine-learning model development. Additionally, 29 LRGs were identified from earlier studies (Liu et al., 2022; Hu et al., 2024; Wang J. et al., 2024). Genes were included if previous studies supported their involvement in lactylation regulation, direct protein lactylation, histone-lactylation-responsive transcription, or lactate handling and the lactoylglutathione pathway (Supplementary Material 1).

**TABLE 1:** Detailed information on datasets used in the study.

DataSets	Platforms	Sample size		Organism	Tissue	Attribute
		Control	AS			
GSE100927	GPL17077	35	69	*Homo sapiens*	Carotid, femoral and infra-popliteal territories	Training
GSE41571	GPL570	6	5	*Homo sapiens*	Carotid intima	Training
GSE28829	GPL570	13	16	*Homo sapiens*	Carotid intima	Validation
GSE43292	GPL6244	32	32	*Homo sapiens*	Carotid intima	Validation

### 2.5 Differential expression analysis

We performed differential expression analysis on the GEO composite dataset as described in Section 2.5 using the “limma” package (Ritchie et al., 2015) in R (version 4.4.1). For this analysis, we set a significance threshold at a corrected *p*-value of less than 0.05 and |Log2Fold Change| greater than 0.4. For the proteomics data, we analyzed quantitative results obtained from Spectronaut and conducted differential protein expression analysis under various conditions using an unpaired t-test, with the filtering criteria set at a *p*-value of less than 0.05 and |Log2Fold Change| greater than 0.4. We visualized the results using the “ggplot” package, creating volcano plots and heatmaps to display the top 50 DEGs. Additionally, we used the “clusterProfiler” package to conduct GO and KEGG enrichment analysis on these genes, aiming to elucidate the biological functions and signaling pathways involved.

### 2.6 Identification of subtypes via consensus clustering

We utilized the “ConsensusClusterPlus” R package for unsupervised clustering analysis (Wilkerson and Hayes, 2010). The K-means algorithm and Euclidean distance served as the foundational clustering methods, analyzing 80% of the sample set and iterating 50 times. We determined the optimal k-value through the consensus cumulative distribution function (CDF) graph and delta area plot. Following this, we produced a heatmap of the consensus clustering with the “pheatmap” R package. To validate the identified two subtypes, we performed principal component analysis (PCA). Furthermore, we assessed the differential expression of lactation-related genes between these subtypes using box plots.

### 2.7 Weighted correlation network analysis

Weighted gene co-expression network analysis was performed using the WGCNA R package (Langfelder and Horvath, 2008). After averaging duplicate probes, the top 25% most variable genes ranked by standard deviation were retained for network construction. Sample quality was assessed using the goodSamplesGenes function, and hierarchical clustering was used to detect outlier samples, with a cut height of 60 and a minimum cluster size of 10. The soft-thresholding power was determined using the pickSoftThreshold function across candidate powers from 1 to 20, based on the scale-free topology fit index and mean connectivity, and β = 19 was selected for subsequent analysis. The adjacency matrix was then transformed into a topological overlap matrix, followed by TOM-based hierarchical clustering. Gene modules were identified using the dynamic tree cut algorithm with deepSplit = 2, pamRespectsDendro = FALSE, and a minimum module size of 60. Module eigengenes were calculated, and module–trait correlations together with gene significance and module membership were used to identify disease-associated modules and hub genes.

### 2.8 Development and validation of diagnostic models through ensemble machine learning algorithms

In our study, we initially performed an intersection analysis of datasets from the GEO database, differentially expressed genes from proteomics, and key module genes from WGCNA analysis to identify candidate diagnostic genes associated with LRGs. To develop a diagnostic model with high accuracy and stability, we then focused on key hub genes. We employed an integrated approach utilizing 12 machine learning algorithms and 113 algorithm combinations, including Random Forest (RF), LASSO regression, stepwise generalized linear models (Stepglm), Support Vector Machine (SVM), partial least squares regression (plsRglm), Naive Bayes, Linear Discriminant Analysis (LDA), generalized linear model boosting (glmBoost), Elastic Net (Enet), Ridge Regression, Extreme Gradient Boosting (XGBoost), and Gradient Boosting Machine (GBM). Initially, we characterized the expression profiles of candidate hub genes associated with LRGs. Subsequently, using a merged dataset from GSE100927 and GSE41571, we executed the 113 algorithm combinations and conducted ten-fold cross-validation to refine diagnostic models. We validated the performance of all models on the GSE28829 and GSE43292 datasets and assessed the area under the curve (AUC) for each model. The model with the highest average AUC was selected as the prime candidate for the potential diagnostic model, revealing four hub genes of the highest diagnostic value. Moreover, we evaluated the top-performing model using a confusion matrix. Finally, to illustrate the model’s effectiveness visually, we created bar charts and line graphs, demonstrating the predictive performance using the rms and rmda packages for bar graphs, calibration curves, and decision curves.

### 2.9 SHAP explainability model

In this research, we employed SHAP values to analyze the relationship between core genes and disease. SHAP is an explainability model grounded in game theory, offering a quantitative assessment of each feature’s contribution to the prediction outcome. Specifically, SHAP values quantify the independent contribution of each core gene to disease risk prediction by accounting for the interactions between features and model predictions. This method addresses the limitations of traditional feature importance assessments, providing a more precise and granular analysis of feature impacts.

### 2.10 Gene set variation analysis (GSVA)

GSVA, a non-parametric and unsupervised technique, is utilized to assess variations in the activity levels of specific gene sets within samples. This method applies kernel smoothing to gene expression data, calculating the relative rankings of genes in each sample. These rankings are then used to quantify gene set activity through kernel estimation techniques. This analysis facilitates the comparison of gene set expression changes across samples, thus uncovering crucial biological pathway activities under various biological conditions.

### 2.11 Gene set enrichment analysis (GSEA)

GSEA is a computational technique used to determine if a specific set of genes is significantly enriched either at the top or bottom of a ranked gene expression list. The process begins by ranking genes according to their correlation coefficients with phenotypic characteristics. It then evaluates the enrichment of the gene set by computing an enrichment score (ES), which reflects the cumulative distribution of the gene set throughout the ranked list. This method not only aids in exploring biological pathways and functional categories within gene expression data but also unveils the potential roles of these pathways across different biological states or disease conditions.

### 2.12 Immune infiltration analysis

To further investigate the immune system changes in patients with AS, this study employed the CIBERSORT R package and the LM22 gene signature, available through the CIBERSORT website, to quantitatively analyze 22 types of immune cells (Bindea et al., 2013). CIBERSORT, a computational method for analyzing expression profile data, accurately estimates the relative abundance of diverse cell types from complex tissue samples. This method relies on linear support vector regression to directly analyze cellular components from tissue-derived gene expression data. In this analysis, we quantified the percentage of each immune cell type in the samples and assessed the differences in these 22 immune cell types between the control and case groups. Additionally, we examined the correlations between these immune cells and LRGs, and visualized the results of the analysis.

### 2.13 Single-cell RNA sequencing analysis

We obtained scRNA-seq data from three AS patients from the GEO database (GSE159677), derived from calcified cores of atherosclerotic plaques and adjacent proximal carotid tissues. The data were processed using the “Seurat” software package (Stuart et al., 2019). To ensure data quality, we conducted quality control on the cells, discarding those that met the following criteria: fewer than 300 or more than 10,000 detected genes, less than 2,000 or more than 25,000 unique molecular identifiers, or cells with mitochondrial gene expression exceeding 15%. Additionally, we removed low-expression genes present in fewer than 3 cells to enhance the reliability of our analysis. We then normalized the filtered data using the LogNormalize method and identified 2,000 highly variable genes via the FindVariableFeatures function. We scaled the data using a scale factor of 10,000 to counteract sequencing depth discrepancies. Subsequently, we performed PCA on the scaled data to reduce dimensions and discern the primary trends in cellular changes. To further elucidate the underlying structural relationships between cells, we conducted UMAP dimension reduction using the RunUMAP function and annotated cell types using the “SingleR” package, ensuring the accuracy and biological relevance of cell classification. Additionally, to examine cellular differentiation trajectories and dynamic changes in gene expression, we employed Monocle 3 for pseudotime analysis, sequencing cells by their developmental states. Finally, we used CellChat software (version 1.6.1) to infer intercellular communication networks (Jin et al., 2021). CellChat, leveraging single-cell expression data, identifies ligand-receptor interactions among different cell types and elucidates potential signaling pathways. In this study, we utilized CellChat to analyze ligand-receptor pairing on the expressed genes of each cell type, constructing a comprehensive cellular communication network and further exploring signaling and regulatory mechanisms between cells.

### 2.14 CMap analysis

CMap (https://clue.io/) is a gene expression database that catalogues cellular responses to various perturbations or interventions (Subramanian et al., 2017). It is extensively utilized to identify potential therapeutic compounds associated with specific diseases. In this study, we integrated the top 100 upregulated DEGs in AS into the CMap database. Subsequently, we identified the top 10 compounds with the highest enrichment scores, which are considered potential therapeutic agents for treating AS.

### 2.15 Molecular docking analysis and molecule dynamics

The molecular structures of ten compounds were retrieved from the PubChem database (https://pubchem.ncbi.nlm.nih.gov/). Additionally, the three-dimensional molecular structures of four key hub genes were obtained from the RCSB PDB database (https://www.rcsb.org/). Molecular docking was executed using AutoDock Vina software, followed by three-dimensional visualization with PyMOL software. A lower docking score indicates higher binding affinity and stability between the ligand and protein. Furthermore, we performed atomic MD simulations using the GROMACS software. The simulation conditions included constant temperature and pressure, along with periodic boundary conditions. The protein and molecule topologies were defined using the AMBER99SB and GAFF force fields, respectively. Simulations were conducted at a temperature of 298 K and a pressure of 1 bar, with 100 ps of NVT and NPT equilibration. Subsequently, a 100 ns molecular dynamics simulation was carried out, with simulation data saved every 10 ps. The binding free energy between the protein and ligand was calculated using the MM/PBSA method (Valdés-Tresanco et al., 2021).

### 2.16 Experimental animals and grouping

The experimental protocol was approved by the Animal Ethics Committee of Jiangxi University of Chinese Medicine (Ethics Number: 20240312025), and the animal experimentation facility was licensed (License Number: SYXK (Gan) 2022-0002). The experimental subjects consisted of 12 male, 8-week-old SPF-grade C57BL/6J mice and 12 male, 8-week-old SPF-grade APOE knockout mice (C57BL/6J strain), each weighing approximately 20 g. All mice were obtained from the Experimental Animal Center of Sipofu (Beijing) Biotechnology Co., Ltd. (License Number: SCXK (Jing) 2024-0001). The animals were housed in an SPF-grade laboratory at Jiangxi University of Traditional Chinese Medicine, with *ad libitum* access to food and water, and were maintained under a 12-h light/dark cycle (25 °C ± 1 °C) for 1 week to acclimate.

The animals were divided into two groups: C57BL/6J mice as the wild-type (WT) control group (n = 12) and Apoe^−/−^ mice as the Apoe^−/−^ group (n = 12). The WT control group was fed a standard diet, whereas the Apoe^−/−^ group was switched to a high-fat diet after 1 week of acclimatization and maintained on this diet for 12 weeks to induce atherosclerotic lesions.

### 2.17 RNA extraction and quantitative real-time PCR

Total RNA was extracted from tissue samples using the Molpure® Cell/Tissue Total RNA Kit (YEASEN) according to the manufacturer’s protocol. RNA quality was assessed and treated with gDNA Eraser (TaKaRa) to remove genomic DNA. cDNA synthesis was performed using the PrimeScript RT Reagent Kit (TaKaRa). Quantitative PCR was conducted on a QuantStudio™ 3 Real-Time PCR System (Thermo Fisher) using TB Green Premix Ex Taq™ II (TaKaRa) with gene-specific primers. The amplification conditions were as follows: 95 °C for 30 s, 95 °C for 5 s, 55 °C for 30 s, and 72 °C for 30 s, with 45 cycles. Relative gene expression was calculated using the 2^−ΔΔCT^ method, normalizing to β-actin as the internal control. The specific primers used in these RT-qPCR assays are detailed in Supplementary Material 2.

### 2.18 Western blotting

Total protein was extracted from mouse aortic tissue using RIPA lysis buffer containing protease inhibitor and PMSF. The homogenates were incubated on ice for 30 min and centrifuged at 12,000 rpm for 10 min at 4 °C, and the supernatants were collected for storage at −80 °C. Protein concentration was determined using a BCA kit, and equal amounts of protein were denatured in 5× loading buffer at 95 °C for 10 min. Proteins were separated by SDS-PAGE on 10% polyacrylamide gels and transferred onto PVDF membranes. After blocking with 5% non-fat milk in TBST for 2 h, the membranes were incubated overnight at 4 °C with primary antibodies against β-actin (1:50,000, Abclonal), Neuropilin-1 (1:2,000, Huabio), and UAP1 (1:2,000, Huabio). After washing, membranes were incubated with HRP-conjugated goat anti-rabbit or goat anti-mouse secondary antibodies (1:8,000, Abclonal) for 2 h at room temperature. Protein bands were visualized using an ECL substrate and imaged with a Tanon 5200 chemiluminescence system. Band intensities were quantified using ImageJ software and normalised to β-actin.

### 2.19 Immunofluorescence staining

Immunofluorescence staining was performed to detect the localisation and expression of NRP1 and UAP1 in mouse aortic tissue. Paraffin sections were deparaffinised, rehydrated through graded ethanol, and subjected to heat-induced antigen retrieval in citrate buffer (microwave, ∼20 min). After cooling, sections were rinsed in PBS and blocked with 3% bovine serum albumin (BSA) for 30 min at room temperature. Sections were then incubated overnight at 4 °C with primary antibodies against NRP1 (1:100, Huabio) and UAP1 (1:100, Huabio). Following PBS washes, sections were incubated with species-specific secondary antibodies conjugated to different fluorophores (e.g., Alexa Fluor 488 goat anti-rabbit and Alexa Fluor 594 goat anti-mouse; 1:100, Servicebio) for 30 min at 37 °C, counterstained with DAPI, and mounted with anti-fade mounting medium. Negative controls without primary antibody were included to confirm staining specificity. Fluorescence signals were visualised and imaged using a fluorescence microscope.

### 2.20 Statistical data analysis

Statistical analysis was performed using GraphPad Prism 10 software. As the data followed a normal distribution and exhibited homogeneity of variance, an independent samples t-test was applied to assess differences in continuous variables between the two groups. Spearman’s correlation analysis was used to evaluate the relationships between different molecules. All statistical tests were two-tailed, with a significance level of *p* < 0.05 considered statistically significant.

## 3 Results

### 3.1 Identification and enrichment analysis of 79 AS-associated genes through proteomics and transcriptomics data

To provide a clearer overview of the discovery analysis, this section integrates two types of data: DIA proteomic data generated from ox-LDL-treated HUVECs and untreated control HUVECs, and the batch-corrected merged transcriptomic cohort composed of GSE100927 and GSE41571, including 74 AS samples and 41 healthy control samples. We first evaluated the quality and overall characteristics of the proteomic data, and then identified common AS-related genes by integrating proteomic and transcriptomic differential expression results.

**FIGURE 2:**
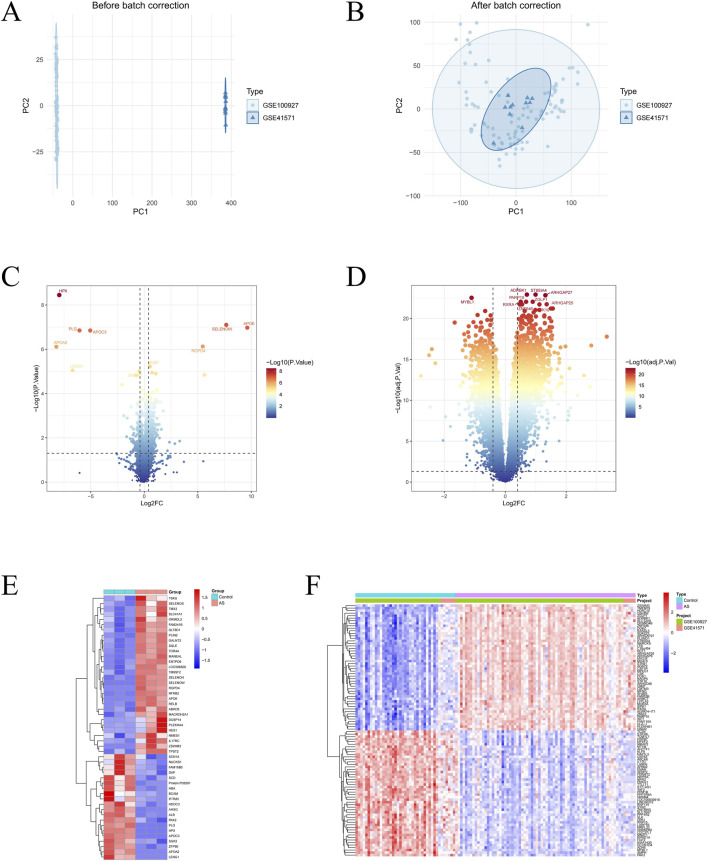
Identification of differentially expressed protein (DEPs) and differentially expressed genes (DEGs). **(A,B)** Two-dimensional PCA cluster plot of GSE100927 and GSE41571 datasets before and after normalization. **(C,D)** Volcano plot of DEPs and DEGs. Red spots represent upregulated genes and blue spots represent downregulated genes. **(E,F)** Heatmap of DEPs and DEGs.

For the proteomics dataset, we performed statistical analyses of peptide length distribution and peptide counts per protein to evaluate the basic characteristics and overall quality of the data (Supplementary Material 3). Based on these data, we identified 472 differentially expressed proteins (DEPs), including 280 upregulated proteins and 192 downregulated proteins. Volcano plots and heatmaps were used to visualize these proteomic differences (Figures 2C, E).

**FIGURE 3:**
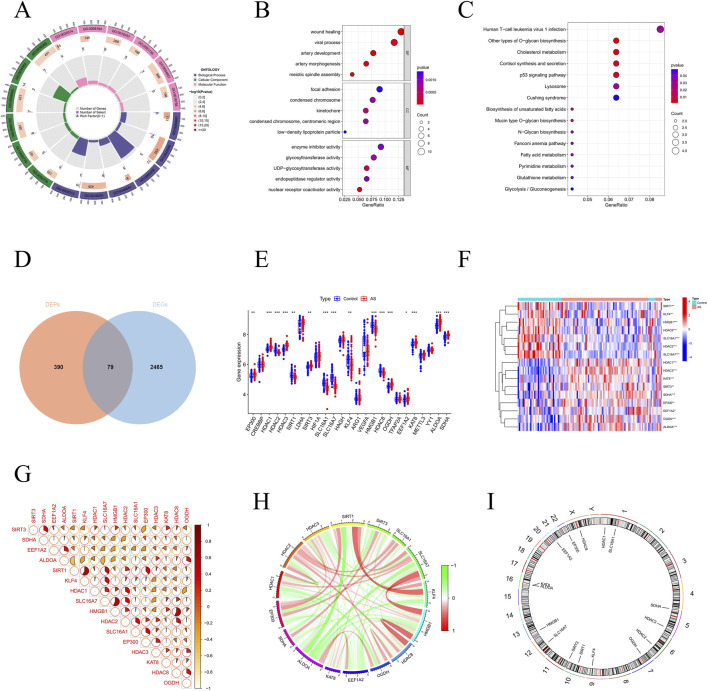
Enrichment analysis and identifying differentially expressed LRGs. **(A,B)** Enriched Gene Ontology (GO) analysis items. “BP,” biological processes; “CC,” cellular component; “MF,” molecular functions. **(C)** Enriched items from the Kyoto encyclopedia of genes and genomes (KEGG) pathway analysis items. **(D)** Venn plot of common genes between DEPs and DEGs. **(E,F)** Overall expression landscape of LRGs in AS. *P < 0.05; **P < 0.01; ***P < 0. 001. **(G,H)** Correlation analysis diagram of 16 differentially expressed LRGs. **(I)** Expression of 16 differentially expressed LRGs at various positions on human chromosomes, with each position marked by a specific gene.

For the transcriptomic dataset, after merging GSE100927 and GSE41571 and correcting for batch effects, we obtained a discovery cohort containing 74 AS samples and 41 healthy controls. Principal component analysis (PCA) before and after batch correction was used to assess the effectiveness of data integration (Figures 2A, B). Using the predefined threshold, we identified 2,544 differentially expressed genes (DEGs), including 1,471 upregulated genes and 1,073 downregulated genes (Figures 2D, F). By intersecting the DEPs and DEGs, we finally identified 79 common AS-related genes shared by the proteomic and transcriptomic analyses (Figure 3D).

We then performed GO and KEGG enrichment analyses on these 79 genes. GO analysis showed that biological processes were mainly enriched in regulation of wound healing, artery development, and artery morphogenesis. In terms of cellular components, these genes were primarily enriched in focal adhesion, condensed chromosome kinetochore, and low-density lipoprotein particle, while molecular functions were mainly associated with enzyme inhibitor activity, glycosyltransferase activity, and regulation of endopeptidase activity (Figures 3A, B). KEGG pathway analysis further indicated enrichment in Human T-cell leukemia virus 1 infection, cholesterol metabolism, cortisol synthesis and secretion, and several biosynthetic pathways (Figure 3C).

### 3.2 Distribution and correlation analysis of differentially expressed LRGs in AS group and control group

We analyzed the expression of 29 LRGs in the merged dataset, revealing significant expression differences between 16 LRGs when comparing the control and AS groups (Figures 3E, F). Specifically, the genes EP300, HDAC1, HDAC3, SIRT3, OGDH, EEF1A2, KAT8, ALDOA, and SDHA were highly expressed in the AS group. Conversely, the genes HDAC2, SIRT1, SLC16A1, SLC16A7, KLF4, HMGB1, and HDAC8 exhibited higher expression levels in the control group. The chromosomal locations of these 16 differentially expressed LRGs were illustrated using a Circos plot (Figure 3I). Additionally, Spearman correlation analysis indicated significant interactions among these differentially expressed LRGs (Figures 3G, H).

### 3.3 Consensus clustering analysis based on DELRGs expression in AS

**FIGURE 4:**
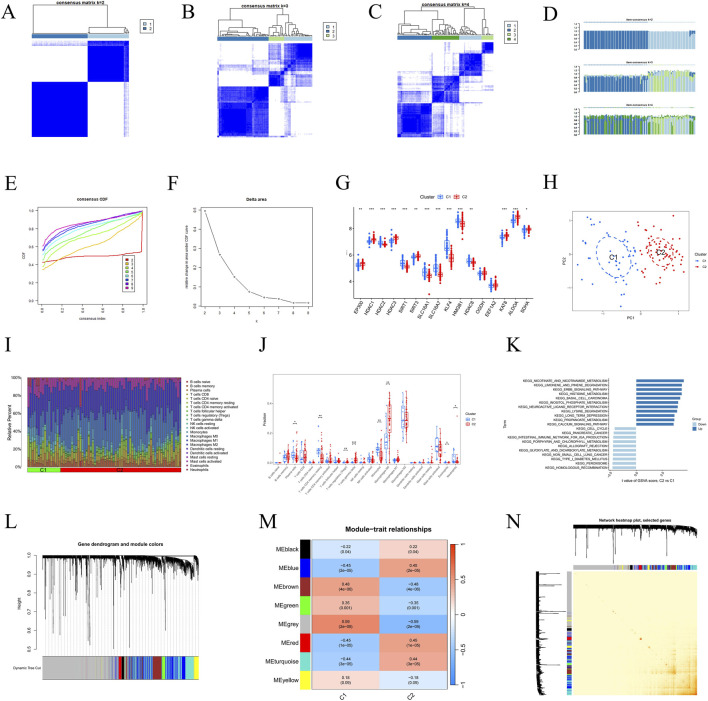
Establishing consensus clustering analysis and analysis of immune infiltration, enrichment analysis and weighted correlation network analysis (WGCNA) between clusters. **(A–C)** Consensus clustering matrixes were generated for values of k ranging from 2 to 4. **(D)** Variation in item consensus values for k = 2, k = 3, and k = 4. **(E)** CDF curves displayed consensus distributions from k = 2 to k = 9. **(F)** Relative change in area under the cumulative distribution function curve for consensus clustering, with k values ranging from 2 to 9. **(G)** The box plot displays the expression patterns of MRGs across two AS clusters *P < 0.05; **P < 0.01; ***P < 0.001. **(H)** This principal component analysis plot shows the distribution of samples based on C1 and C2. **(I)** Histogram showing the proportion of immune cells within the samples. **(J)** Comparison of immune cell proportions between categories C1 and C2. **(K)** Display of t-values for GSVA scores comparing C2 versus C1 based on KEGG enrichment analysis. **(L)** Gene dendrogram illustrating the hierarchical clustering of genes and their corresponding module colors. **(M)** Heatmap illustrating interactions among selected genes within a network. **(N)** Heatmap showing the module-trait relationships.

To investigate lactylation modification patterns in AS, we performed consensus clustering analysis on AS patients from the merged dataset. We set the number of clusters (K) to 2, observing that the cumulative distribution function (CDF) plot indicated the narrowest consensus index range from 0.1 to 0.9, with the delta region scoring highest (Figures 4E, F). The consensus matrix for K = 2 demonstrated the greatest consistency (Figures 4A–D), leading to the classification of AS patients into two distinct subtypes, termed Subtype 1 and Subtype 2. Further validation through principal component analysis confirmed the separation between these subtypes, revealing distinct clustering along the first two principal component axes (Figure 4H). This supports the robustness of our clustering results. Box plot analyses showed that seven differentially expressed large regulatory genes (DELRGs), namely, HDAC2, SIRT1, SLC16A1, SLC16A7, KLF4, HMGB1, and HDAC8, were significantly expressed in Subtype 1. Conversely, another set of seven DELRGs, including EP300, HDAC1, HDAC3, SIRT3, KAT8, ALDOA, and SDHA, exhibited significant expression in Subtype 2 (Figure 4G). The distinct expression patterns of these genes in the subtypes underscore their varied functional roles in AS.

### 3.4 Identification of biological functional features and immune microenvironment in different subgroups

We conducted an analysis to compare the immune microenvironment characteristics of the two subtypes. The findings revealed that Subtype 1 exhibited high levels of immune infiltration, including Plasma cells, resting memory CD4^+^ T cells, Monocytes, Eosinophils, and Neutrophils. In contrast, Subtype 2 showed significantly higher infiltration of regulatory T cells (Tregs), gamma delta T cells, and M0 Macrophages (Figures 4I, J). Additionally, we used GSVA to evaluate key biological pathway activities under various conditions for each subtype. In Subtype 1, pathways such as peroxisomal metabolism and glyoxylate and dicarboxylate metabolism were significantly activated. Conversely, Subtype 2 displayed significant activation in pathways like nicotinate and nicotinamide metabolism, histidine metabolism, and the ERBB signaling pathway (Figure 4K).

### 3.5 Acquisition of candidate hub genes

We applied WGCNA to identify the modules with the highest connectivity across the two subtypes. Initially, we set the cut height (h) to 60, excluded two outlier samples (GSM2696641 and GSM2696632), and retained 103 samples for analysis (Supplementary Material 4A). Candidate soft-thresholding powers ranging from 1 to 20 were then evaluated using the scale-free topology fit index and mean connectivity, and the optimal soft-thresholding power was determined to be β = 19. (Supplementary Material 4B). Clustering analysis revealed eight modules, with the grey module showing the highest correlation coefficient (MEgrey: r = 0.59, p = 2e−09) (Figures 4L–N). By intersecting the 2,059 genes from the grey module with 79 previously identified AS-related genes, we ultimately identified 25 candidate hub genes with potential diagnostic significance (Supplementary Material 4C).

### 3.6 Identification of hub genes with diagnostic value through machine learning models

**FIGURE 5:**
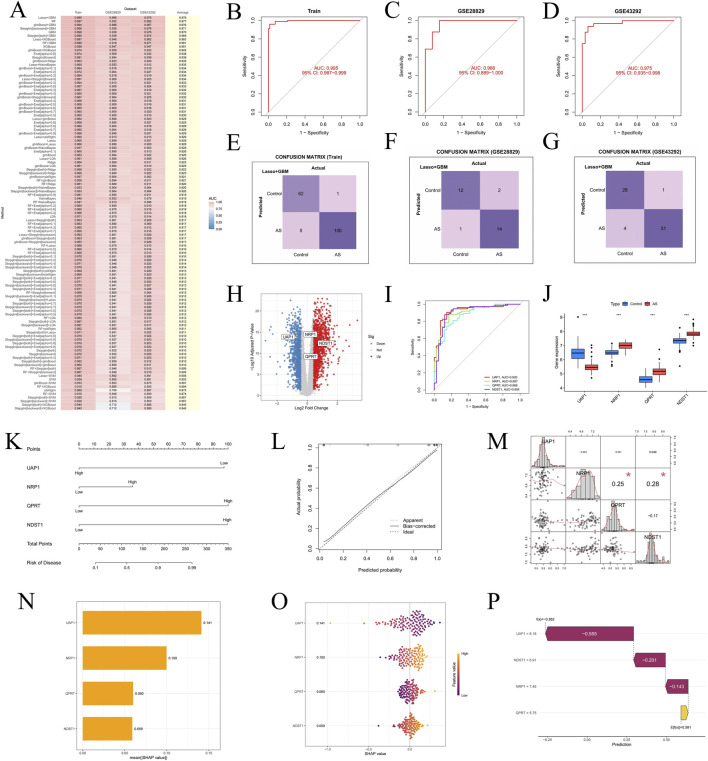
Identify hub genes associated with LRGs in two AS groups using a machine learning diagnostic model and acquisition and validation of hub genes. **(A)** Heatmap showing the performance of different machine learning diagnostic models in the training set and in the two external validation datasets (GSE28829 and GSE43292), as evaluated by the AUC values. **(B–D)** ROC curves of the selected diagnostic model in the training set, GSE28829, and GSE43292, respectively, demonstrating its predictive accuracy and robustness in distinguishing the two AS groups. **(E–G)** Confusion matrices illustrate the model’s predictive performance on the training dataset and external datasets GSE28829 and GSE43292. **(H)** Volcano plot highlighting four key hub genes. **(I)** ROC curve analysis demonstrates the diagnostic accuracy of the four hub genes in the training dataset. **(J)** Box plots comparing the expression levels of the four hub genes between control and treatment groups. **(K,L)** Nomogram developed for clinical use, enabling precise prediction of AS risk based on the expression of hub genes. **(M)** Correlation matrix chart depicting the interrelationships among the four hub genes, with each cell showing the Pearson correlation coefficient. Significance levels are indicated as *P < 0.05; **P < 0.01; ***P < 0.001. **(N)** SHAP bar chart. **(O)** SHAP beeswarm plot. **(P)** SHAP waterfall plot.

In this study, we used a merged dataset as the training set and GSE28829 and GSE43292 as validation sets to construct a diagnostic model. We tested 12 machine learning algorithms with 10-fold cross-validation, generating a total of 113 combination models to evaluate 25 candidate hub genes. The average AUC for each model was calculated for both training and validation datasets. The LASSO + GBM algorithm emerged as the optimal model, achieving AUC values of 0.995, 0.966, and 0.975 in the training and validation sets, respectively, with an overall average AUC of 0.979 (Figures 5A–D). Furthermore, the accuracy and robustness of the LASSO + GBM model were validated using confusion matrix plots (Figures 5E–G). This model identified four hub genes (UAP1, NRP1, QPRT, and NDST1) with the highest diagnostic significance, and corresponding volcano plots were generated (Figure 5H).

### 3.7 Analysis of hub gene expression for AS diagnostic efficacy and construction of disease risk prediction models

We evaluated the diagnostic efficacy of the four hub genes in AS using ROC curves, where the x-axis represents 1-specificity and the y-axis represents sensitivity. The graph presents the AUC values for each gene: UAP1 (0.920), NRP1 (0.897), QPRT (0.868), and NDST1 (0.904), highlighting their diagnostic potential. Notably, UAP1 and NDST1, with AUC values above 0.9, demonstrate particularly strong diagnostic accuracy (Figure 5I). Box plot analysis further illustrates significant differences in the expression levels of these hub genes between disease and control groups. Specifically, NRP1, QPRT, and NDST1 show significantly higher expression in the disease group, while UAP1 is more highly expressed in the control group (Figure 5J). Additionally, gene expression density distribution and correlation analyses reveal a significant positive correlation between QPRT and NDST1, whereas UAP1 and NRP1 show a weaker correlation. These findings offer important insights for further exploration of the biological roles of these genes in AS (Figure 5M).

Additionally, we developed a nomogram based on the four hub genes to predict AS disease risk. Each gene’s expression level corresponds to specific risk scores; as the cumulative score increases, the predicted disease risk also rises, reaching a maximum value of 0.99 (Figure 5K). Furthermore, the calibration curve indicates high concordance between the model’s predicted probabilities and the actual outcomes, with the corrected bias curve closely aligning with the ideal curve, further supporting the model’s accuracy and reliability in predicting disease risk (Figure 5L).

To further explore the relationship between core gene expression and disease prediction, and to analyze the contribution of individual features to the single-sample prediction results, we integrated three datasets (GSE100927, GSE43292, and GSE41571) and removed batch effects (Supplementary Material 5). The integrated dataset was divided into a training set (70%) and a test set (30%), and ten machine learning models were employed for training and evaluation. ROC curve analysis indicated that the Partial Least Squares (PLS) model exhibited the best predictive performance in this study, with an AUC value of 0.922 (Supplementary Material 6). To further investigate the relationship between core gene expression and disease prediction, we conducted SHAP value analysis on the most important genes in the model to assess their contribution to the single-sample prediction results. A bar chart displayed the ranking of genes based on their SHAP values, with UAP1 having the highest SHAP value (0.141), indicating its significant impact on disease prediction. NRP1 followed with a SHAP value of 0.100, showing its substantial contribution. In contrast, the SHAP values for QPRT and NDST1 were relatively low—0.060 and 0.059, respectively—suggesting that these two genes had a smaller effect on the prediction results (Figure 5N). Additionally, a swarm plot illustrated the distribution of SHAP values for these genes, showing that higher expression of UAP1 was typically associated with lower SHAP values, implying that its elevated expression contributes to predicting the disease state. Similarly, NRP1 and QPRT displayed a similar pattern, where higher gene expression values were correlated with lower SHAP values (Figure 5O). Furthermore, a waterfall plot presented the SHAP value analysis results for individual samples, emphasizing the significant roles of UAP1 and NDST1 in model predictions. Specifically, UAP1 and NDST1 had a notable negative impact on the prediction results, with SHAP values of −0.555 and −0.201, respectively (Figure 5P). Overall, the expression changes in UAP1 had the greatest impact on the model’s prediction results, while QPRT contributed relatively little to the prediction.

### 3.8 GSEA and GSVA analysis of hub genes

**FIGURE 6:**
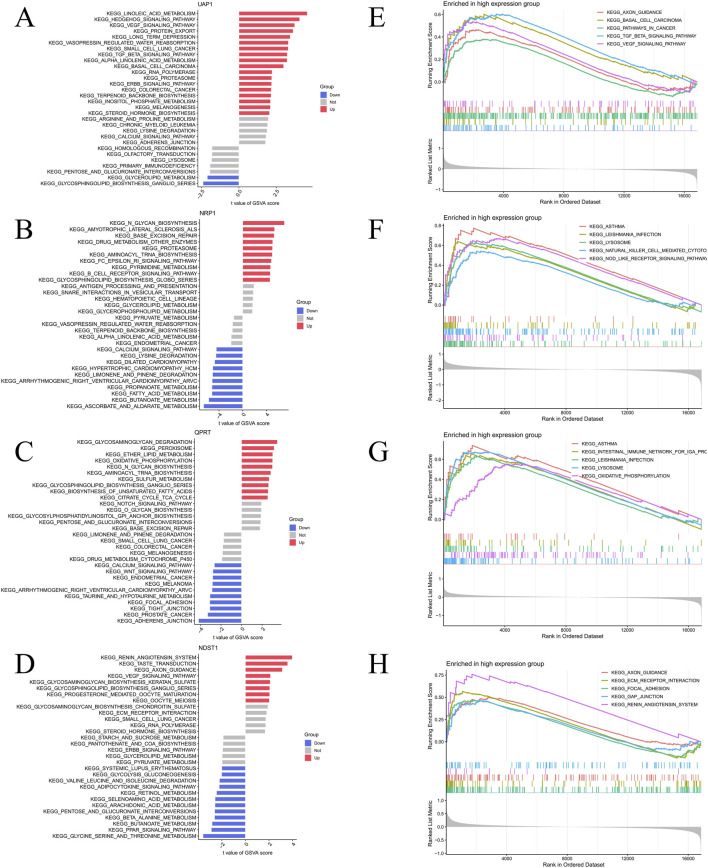
GSVA and GSEA of hub genes. **(A–D)** GSVA results for the hub genes UAP1, NRP1, QPRT, and NDST1, showing the signaling pathways potentially associated with each gene based on variation in pathway activity across samples. **(E–H)** GSEA results for the hub genes UAP1, NRP1, QPRT, and NDST1, illustrating the significantly enriched signaling pathways associated with high expression of each hub gene.

To further investigate the biological functions of the four key genes and their involvement in signaling pathways, we performed GSEA and GSVA. GSEA results show that UAP1 is primarily enriched in pathways such as axon guidance, extracellular matrix-receptor interaction, and the renin-angiotensin system (Figure 6A). NRP1 is significantly involved in pathways related to lysosomes, natural killer cell-mediated cytotoxicity, and NOD-like receptor signaling (Figure 6B). QPRT is associated with processes like intestinal immune network IgA production, lysosomal function, and oxidative phosphorylation (Figure 6C). NDST1 is mainly enriched in axon guidance, TGF-β signaling, and the VEGF signaling pathway (Figure 6D). GSVA reveals that high UAP1 expression is significantly associated with pathways such as linoleic acid metabolism, protein transport, VEGF signaling, and TGF-β signaling (Figure 6E). High NRP1 expression upregulates critical signaling pathways including N-glycan biosynthesis, aminoacyl-tRNA biosynthesis, B cell receptor signaling, and glycosphingolipid biosynthesis (Figure 6F). Similarly, high QPRT expression shows significant enrichment in ether lipid metabolism, oxidative phosphorylation, N-glycan biosynthesis, and glycosphingolipid biosynthesis (Figure 6G). NDST1 expression is also significantly enriched in the renin-angiotensin system, VEGF signaling, and glycosphingolipid biosynthesis (Figure 6H).

### 3.9 Analysis of AS immune cell lineages and the association with key genes

**FIGURE 7:**
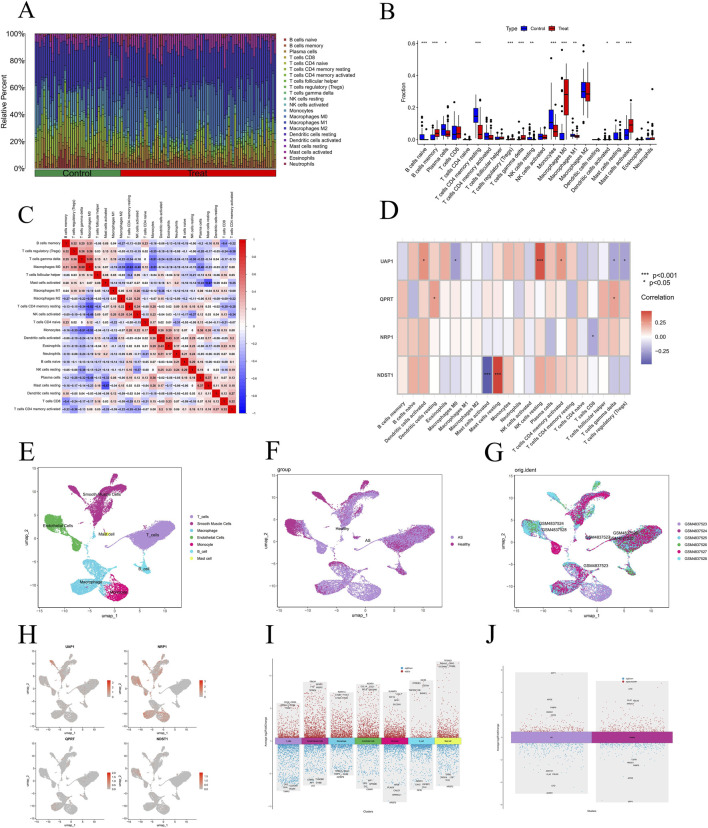
Analysis of immune infiltration in AS and analysis of single-cell RNA sequencing data from GSE159677. **(A)** Histogram depicting the proportions of various immune cells. **(B)** Comparative analysis of immune cell proportions between AS patients and control subjects. **(C)** Heatmap illustrating the correlation analysis among different immune cell subtypes. **(D)** Detailed correlation analysis involving 22 types of immune cells in AS, displayed through a heatmap and a cell association graph to show the relationships with the expression of key hub genes. **(E)** UMAP plot showing the distribution of cell types, each cell type is colored differently. **(F)** UMAP distribution of cells from patients and healthy controls, with cells from AS tissue in purple and normal tissue in pink. **(G)** UMAP plot categorized by sample IDs showing cell distribution, with different colors representing different samples. **(H)** UMAP plot shows the expression distribution of selected hub genes across different cell types. **(I)** Displays significant upregulation and downregulation of gene expression across various cell clusters. **(J)** Illustrates significant differences in gene expression between AS tissue and normal tissue. Significance levels are indicated as *P < 0.05; **P < 0.01; ***P < 0.001.

In the merged dataset, we analyzed immune cell infiltration levels in patients with AS compared to healthy controls. Among the 22 types of immune cells analyzed, 5 showed significantly elevated infiltration levels in AS patients, while 8 displayed significantly decreased levels (Figures 7A–C). Additionally, we examined the influence of the four key genes on immune cell infiltration. The expression of UAP1 was significantly correlated with the infiltration levels of 6 types of immune cells. Meanwhile, NRP1 expression was significantly negatively correlated with CD8^+^ T cell infiltration. QPRT expression showed a significant positive correlation with gamma delta T cells and resting dendritic cells. Finally, NDST1 expression was significantly positively correlated with resting mast cell infiltration but negatively correlated with active mast cell infiltration (Figure 7D).

### 3.10 Single-cell analysis

**FIGURE 8:**
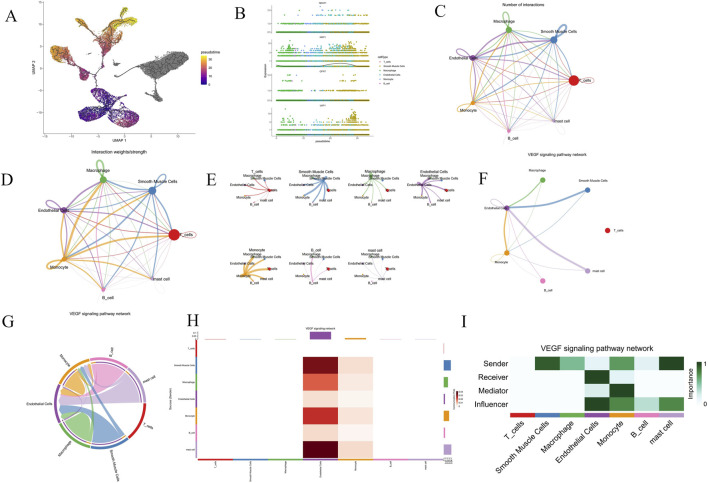
Pseudo-temporal analysis and cell-cell communication analysis. **(A)** Displays the cellular developmental trajectory and pseudotime analysis using UMAP. **(B)** Illustrates the expression levels of four hub genes across various cell types over pseudotime. **(C)** Interaction net count plot of AS cells. The thicker the line, the greater the number of interactions. **(D)** Interaction weight plot of AS cells. The thicker the line, the stronger the interaction weights/strength between the two cell types. **(E)** Detailed network of cell–cell interactions among 7 cell subsets. **(F)** VEGF signaling pathway network diagram showing interactions among various cell types. **(G)** Chord diagram of the VEGF signaling network, detailing the pathways of signal transmission between cells. **(H)** Heatmap of interaction strengths in the VEGF signaling pathway among different cell types. **(I)** Heatmap of cell roles in the VEGF signaling pathway network.

The GSE159677 dataset includes scRNA-seq data from six major cell types within the carotid arteries of three AS patients, covering both calcified plaques and adjacent tissue samples. Following preliminary quality control, the top 2000 highly variable genes were selected for further analysis (Supplementary Material 7A). A resolution of 0.1 was applied for clustering (Supplementary Material 7B). Using the UMAP algorithm for dimensionality reduction, cells were categorized into eight distinct clusters. Cell types were subsequently annotated with the “SingleR” tool, identifying seven types: T cells, smooth muscle cells, macrophages, endothelial cells, monocytes, B cells, and mast cells. Three UMAP plots were generated to display cell distribution by type, group, and sample (Supplementary Material 7C) (Figures 7E-G). Analysis indicated that all hub genes were significantly expressed in macrophages and smooth muscle cells, with NRP1 and NDST1 also highly expressed in endothelial cells and monocytes, and UAP1 prominently expressed in both endothelial cells and T cells (Figure 7H). Differential analysis across the seven cell types and groups was performed, and volcano plots were generated (Figures 7I, J). Using Monocle 3 pseudotime analysis, we mapped the developmental states of cells, employing the UMAP algorithm to visualize cell differentiation trajectories and transitions from early to late stages (Figure 8A). Jitter plots (Supplementary Material 7D) provided detailed expression profiles of selected genes across different cell types along the pseudotime, revealing expression patterns of key genes like TPSAB1 and NRP1 in specific cell types. Differential gene distribution plots (Figure 8B) further highlighted the expression characteristics of four hub genes—UAP1, QPRT, NRP1, and NDST1—in specific cell states, with pronounced expression differences particularly in smooth muscle cells and endothelial cells, underscoring their pivotal roles in AS progression. Additionally, using the CellChat tool, we examined communication patterns among various cell populations, focusing on differentially expressed ligands and receptors to assess intercellular communication potential, and visualized these interactions (Figures 8C–E). Further analysis revealed significant variations in communication patterns via the VEGF signaling pathway across cell types. Analysis of ligand-receptor pair contributions and gene expression data identified critical roles for specific molecular interactions and genes within the VEGF signaling pathway, highlighting the complexity and diversity of intercellular signaling (Figures 8F–I).

### 3.11 Identification of candidate small molecule compounds for the treatment of AS

To identify small molecule compounds with potential therapeutic effects on AS through gene expression regulation, we imported the top 100 upregulated DEGs from AS samples into the CMap database. Analysis identified the ten compounds with the highest negative scores: phenytoin, tubocurarine, ganaxolone, HG-14-10-04, piperine, diflorasone, rivaroxaban, ARC-239, amitifadine, and mesopram. Based on their negative CMap scores, these compounds may counteract the gene expression changes associated with AS, making them promising candidate drugs for AS treatment. The three-dimensional structures of these ten compounds were retrieved from the PubChem database (Supplementary Material 8).

### 3.12 Molecular docking and MD simulation between rivaroxaban and NRP1

**FIGURE 9:**
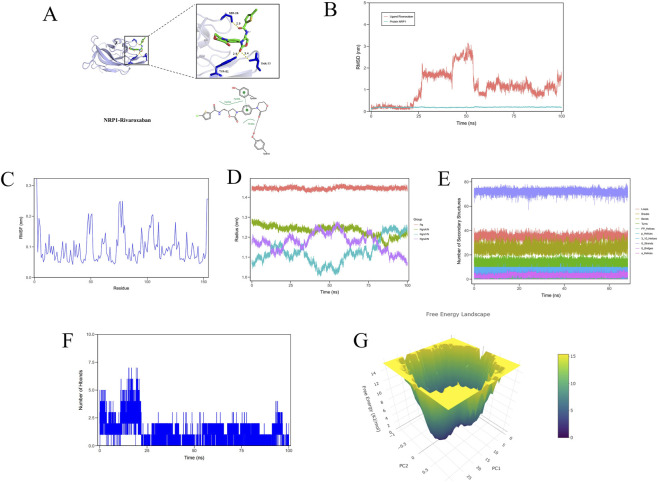
Molecular docking analysis and molecule dynamics. **(A)** A stable interaction between the NRP1 protein and Rivaroxaban was identified. Rivaroxaban engages in hydrogen bonding with the amino acid residues SER26, ASN28, ASP48, SER74, THR77, and TYR81 of NRP1. Additionally, it forms hydrophobic interactions with THR44 and π-stacking with TYR25, contributing to the binding stability. **(B)** RMSD (Root Mean Square Deviation) variations of the protein NRP1 and ligand Rivaroxaban over 100 nanoseconds during molecular dynamics simulations. **(C)** RMSF (Root Mean Square Fluctuation) analysis of protein residues throughout a molecular dynamics simulation. **(D)** Radius of gyration changes of proteins in different groups during molecular dynamics simulations. The x-axis represents the simulation time (nanoseconds), and the y-axis shows the radius of gyration (nanometers). The red line represents the Rg group, green for Rg/S/X, purple for Rg/S/Y, and blue for Rg/S/Z. **(E)** Variation in the number of secondary structures in a protein during a 68-nanosecond molecular dynamics simulation. The x-axis represents time in nanoseconds, while the y-axis shows the count of secondary structures. Different colored layers represent various types of secondary structures, including α-helices, β-strands, β-bridges, 3_10 helices, π-helices, PP helices, turns, bends, breaks, and loops. **(F)** Variation in the number of hydrogen bonds in a protein during a 100-nanosecond molecular dynamics simulation. **(G)** 3D free energy landscape.

Our extensive literature review identified Rivaroxaban as the compound most closely associated with cardiovascular diseases among the ten previously studied small molecules. As a direct inhibitor of factor Xa, Rivaroxaban significantly reduces the risk of stroke and thrombotic events in cardiovascular patients by targeting key stages of the coagulation cascade (Chen et al., 2020; Ma et al., 2021). Studies suggest that NRP1 plays a pivotal role in the vascular lesions and inflammation of AS by modulating angiogenesis and endothelial cell function through VEGF signaling (Domingues and Fantin, 2021). Additionally, lactic acid can promote the expression and release of growth factors like VEGF, which may subsequently influence NRP1 activity and function (Huang et al., 2021). However, no studies have yet reported on the relationship between Rivaroxaban and NRP1. In this study, molecular docking analysis revealed a low docking score of −7.6 kcal/mol, indicating a strong binding affinity between Rivaroxaban and NRP1. Visualization of the docking results demonstrated that Rivaroxaban forms hydrogen bonds with the amino acid residues SER26A, ASN28A, ASP48A, SER74A, THR77A, and TYR81A in NRP1, along with hydrophobic interactions with THR44A and π-stacking with TYR25A (Figure 9A).

We subsequently conducted MD simulations to explore the binding interactions, structural stability, and conformational flexibility between NRP1 and rivaroxaban in greater depth. Throughout the simulation, the root-mean-square deviation (RMSD) of the NRP1 protein remained stable below 0.3 nm with minimal fluctuations, indicating a high level of structural stability. In contrast, the RMSD of rivaroxaban varied between 0.5 and 3 nm, suggesting a series of conformational adjustments within the NRP1 binding pocket (Figure 9B). Root-mean-square fluctuation (RMSF) analysis showed that most regions of the protein had RMSF values below 0.2 nm, indicating high structural rigidity. However, the RMSF values for residues 50 to 100 reached up to 0.2 nm, suggesting significant flexibility in this region, which may be critical for regulating ligand-binding dynamics (Figure 9C). The radius of gyration also fluctuated between 1.0 and 1.4 nm during the simulation, further supporting dynamic adjustments in molecular structure (Figure 9D). Secondary structure analysis revealed stability in α-helices and β-sheets, while the number of turns and loop structures fluctuated, reflecting adaptive structural changes under dynamic conditions (Figure 9E). Additionally, monitoring hydrogen bond counts revealed fluctuations between 0 and 7 bonds between NRP1 and rivaroxaban, with counts exceeding 5 bonds multiple times within the first 25 nanoseconds, indicating strong binding stability. This suggests that hydrogen bonds are crucial in maintaining complex stability (Figure 9F). We also obtained the free energy landscape through PCA, revealing the free energy distribution of the molecular system in the principal conformational space, highlighting the relationship between low free energy regions and the system’s stable conformations (Figure 9G). Finally, binding free energy and energy components, predicted by MM/PBSA, are shown in Table 2. The binding free energy for NRP1/rivaroxaban was calculated to be −26.421 kcal/mol, indicating robust binding stability between the ligand and protein.

**TABLE 2:** Binding free energies and energy components predicted by MM/PBSA (kcal/mol).

System name	NRP1/Rivaroxaban
ΔEvdw	−35.121
ΔEelec	−30.808
ΔGGB	44.162
ΔGSURF	−4.653
ΔGSA	−65.93
ΔGSOLV	39.509
ΔGMM/PBSA	−26.421

ΔEvdW: van der Waals energy; ΔEelec: electrostatic energy; ΔGGB: electrostatic contribution to solvation; ΔGSURF: surface free energy; ΔGSA: non-polar contribution to solvation; ΔGSOLV: solvation free energy; ΔGMM/PBSA: free energy changes calculated by the Molecular Mechanics/Poisson-Boltzmann surface area method.

### 3.13 Validation in animal experiments

**FIGURE 10:**
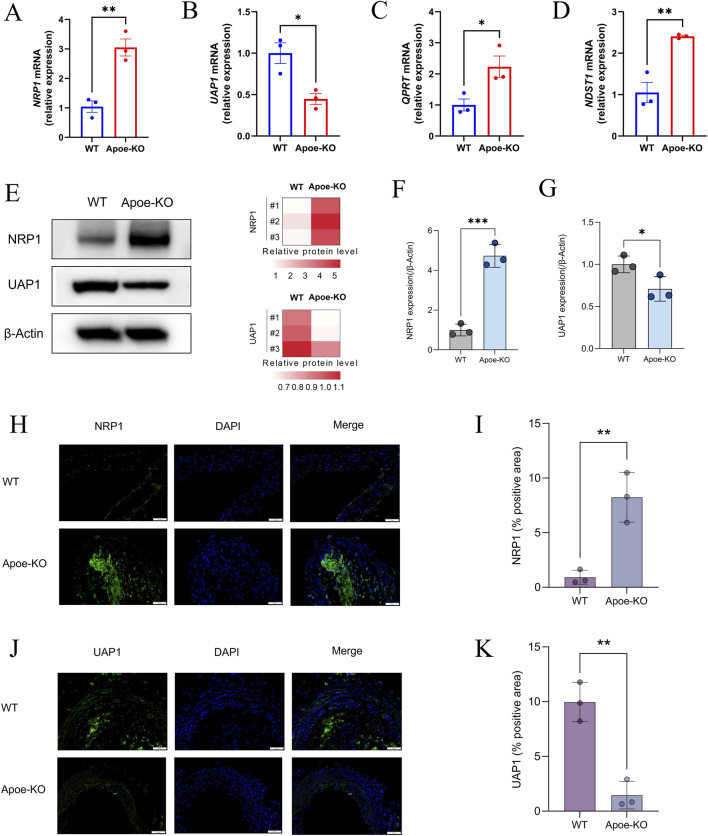
Verification of core genes. **(A–D)** The relative mRNA levels of NRP1, UAP1, QPRT, and NDST1 in AS and control mice, normalized to β-actin. **(E–G)** The relative protein levels of NRP1 and UAP1 in AS and control mice, normalized to β-actin. **(H–K)** Immunofluorescence staining of NRP1 and UAP1 in AS mice and control mice. Significance levels are indicated as *P < 0.05; **P < 0.01; ***P < 0.001.

To interrogate the involvement of lactylation-related genes in AS, we quantified their transcriptional and protein expression in mouse aortic tissue from the WT and Apoe^−/−^ groups. RT-qPCR demonstrated significant differential expression for all four core genes: NRP1, NDST1 and QPRT were upregulated, whereas UAP1 was downregulated in the Apoe^−/−^ group relative to controls (*P* < 0.05), in line with our preceding bioinformatics predictions (Figures 10A–D). To substantiate these findings at the protein level, we assessed NRP1 and UAP1 in mouse aortic tissue by Western blotting and immunofluorescence staining. Both assays confirmed higher NRP1 and lower UAP1 protein abundance in the Apoe^−/−^ group, mirroring the mRNA trends (Figures 10E–I). Collectively, these results reinforce the involvement of NRP1, NDST1, QPRT and UAP1 in AS pathogenesis and prioritise NRP1 and UAP1 for subsequent mechanistic investigation.

## 4 Discussion

AS development encompasses multiple intricate pathophysiological processes, such as smooth muscle cell migration, lipid deposition within arterial walls, and the recruitment of inflammatory cells including monocytes and macrophages (Bonetti et al., 2021; Malekmohammad et al., 2021). Our research demonstrates significant alterations in the expression of various immune cells in AS patients, encompassing B cells, diverse T cell subsets, macrophages of different phenotypes, and dendritic cells. These alterations not only signify the presence of inflammation but also correlate closely with plaque formation and progression. Particularly, a comprehensive analysis of expression data revealed a marked increase in M1 macrophage activity and dendritic cell activation in atherosclerotic conditions. These cells markedly intensify the local inflammatory response by elevating the release of tumor necrosis factor α (TNF-α), interleukin-6 (IL-6), and interleukin-12. This inflammatory process not only fosters the development of the lipid core within plaques but also heightens plaque instability and the risk of rupture through enhanced immune cell activation and increased metalloproteinase expression (Festuccia et al., 2014; Ruytinx et al., 2018; Luo et al., 2024). Moreover, the detection of memory T cells suggests a role for adaptive immune responses in the prolonged progression of AS, potentially linked to chronic inflammation and recurrent endothelial injury (Koltsova et al., 2012; Spitz et al., 2016). These insights underscore the complexity of interactions among immune cells and AS, emphasizing the critical role of immune modulation in its progression.

While traditional therapies such as lipid-lowering, antihypertensive, and antiplatelet treatments effectively manage AS, they fall short in preventing complex late-stage events like cardiovascular accidents. These treatments struggle to control long-term cardiovascular risks due to the inconsistent effects of medications such as Omega-3 fatty acids, whose efficacy varies with dosage and baseline risk levels of study subjects. Additionally, while emerging medications like Bempedoic acid and PCSK9 inhibitors significantly improve LDL cholesterol reduction and enhance efficacy when used in conjunction with statins, these regimes also raise concerns regarding costs and potential adverse effects (Khan et al., 2022; Sucato et al., 2024). Recent research has pivoted to developing more precise targeted therapies, including gene editing technologies to intervene in specific inflammatory pathways and novel biologics that modulate the inflammatory response in vascular endothelium. However, the long-term safety and efficacy of these novel therapies require further verification through clinical trials (Li Z. H. et al., 2023; Jia et al., 2024; Zubirán et al., 2024). Therefore, ongoing research should prioritize the development of novel biomarkers for early diagnosis and disease monitoring, and the exploration of new therapeutic targets to optimize long-term treatment strategies and improve therapeutic outcomes.

In this study, we conducted proteomic analyses through cellular experiments to identify proteins differentially expressed in association with AS. We also analyzed transcriptomic datasets with bioinformatics and machine learning techniques to explore AS subtypes linked to lactylation modifications. Various machine learning methods were used to construct predictive models, identifying four pivotal genes: UAP1, NRP1, QPRT, and NDST1. Furthermore, we explored the possible relationships between these genes and various immune cell lineages. Molecular docking and MD simulations indicated that Rivaroxaban could potentially inhibit NRP1 by forming a stable interaction with its active site. Our findings reveal biomarkers relevant to AS and their roles in metabolic pathways, enhancing our understanding of the disease’s complex pathophysiology and potentially guiding future therapeutic strategies towards personalized medicine.

Lactylation, the covalent attachment of lactate molecules to lysine residues, forms lactylated proteins that are integral to cellular metabolism, signal transduction, and gene expression. As a vital metabolic product, lactate not only contributes to energy supply but also serves as a signaling molecule, influencing neuronal excitability, synaptic plasticity, and various physiological processes, including learning and memory. Within the central nervous system, lactate promotes interneuronal signal transmission via specific transporters and receptors (Liu et al., 2024). Additionally, lactylation regulates gene transcription; notably, histone lactylation induced by lactate significantly increases at the promoters of M2-type genes involved in tissue repair, enhancing their expression. This modification becomes more pronounced under inflammatory conditions, promoting macrophage polarization and anti-inflammatory properties, thus aiding tissue repair (Zhang et al., 2019; Li R. et al., 2023). Recently, lactylation has emerged as a target for clinical applications, with its regulatory mechanisms offering new therapeutic insights in central nervous system disorders, cardiovascular diseases, and metabolic disorders. Importantly, research has demonstrated that lactate amplifies local inflammatory responses by regulating the nuclear factor κB (NF-κB) signaling pathway and enhancing the production of pro-inflammatory cytokines. This response is pivotal in the development of AS, as lactate activates the NF-κB pathway, boosting the production of key inflammatory mediators such as TNF-α and IL-6. Additionally, lactate interacts with G protein-coupled receptor 81 (GPR81), inhibiting the activation of YAP in macrophages. This inhibition diminishes the nuclear translocation of NF-κB, thereby inhibiting the release of pro-inflammatory cytokines and alleviating inflammation associated with AS (Yang et al., 2020; Manosalva et al., 2021). Concurrently, lactylation significantly influences the phenotype of smooth muscle cells, promoting their transition to synthetic and migratory states, critical for intimal thickening of blood vessels (Zhang et al., 2022; Zhou et al., 2023; Zhu et al., 2023). Further studies indicate that lactate accumulation enhances AMPK activity, regulates energy metabolism, and curbs excessive cell proliferation, mechanisms essential for maintaining cellular metabolic balance (Zhou et al., 2022; Keerthana et al., 2023). Additionally, lactylation may impair endothelial cell function and increase vascular permeability, exacerbating AS development (Li et al., 2024). Thus, by impacting cellular functions and vascular integrity, lactylation plays a critical role in the progression of AS and deserves further exploration as a potential therapeutic target. Currently, AS diagnosis involves comprehensive analyses using imaging, blood biomarkers, and clinical evaluations. Early detection is vital for reducing cardiovascular event risks (Gatto and Prati, 2020; Meng et al., 2022). By leveraging multi-omics data and applying machine learning techniques, we have identified key hub genes, such as UAP1, NRP1, QPRT, and NDST1, which may serve as potential biomarkers to distinguish atherosclerotic patients from healthy individuals. UAP1 (UDP-N-acetylglucosamine transferase 1) is a critical membrane-bound enzyme predominantly responsible for the biosynthesis of glycosaminoglycans (GAGs) and glycoproteins. It catalyzes the transfer of UDP-N-acetylglucosamine to various substrates, thereby facilitating the synthesis of GAGs such as hyaluronic acid, heparin, and chondroitin sulfate (Liu et al., 2013; Lai et al., 2019; Wang et al., 2021). These GAGs are essential for cellular signaling, extracellular matrix (ECM) formation, and tissue repair (Shi et al., 2021). UAP1 expression is intricately linked to physiological and pathological processes including cell proliferation, differentiation, and wound healing, with aberrant expression associated with diseases such as cancer and metabolic disorders. Particularly in cardiovascular diseases and AS, UAP1 modulates endothelial cells, smooth muscle cells, and macrophages by regulating GAG synthesis, impacting cell proliferation, migration, and inflammatory responses (Shafi, 2020; Yao et al., 2022). Additionally, UAP1 influences inflammatory pathways, as studies have shown it modulates pro-inflammatory cytokine expression, through the NF-κB signaling pathway, thereby worsening inflammation and fostering the development of atherosclerotic plaques. Abnormal UAP1 expression can disrupt glycosaminoglycan synthesis, affecting the stability and elasticity of blood vessels and accelerating AS development (Kempe et al., 2005; Ding and Chen, 2023). The relationship between UAP1 and lactate, particularly its lactylation modifications, has recently gained attention. Lactate, as a metabolic byproduct, not only impacts cellular functions but also modulates UAP1 expression and activity via the AMPK pathway, altering GAG synthesis (Zhou et al., 2022). Furthermore, lactylation modifications may directly affect UAP1’s catalytic efficiency, impacting its substrate binding capacity and thereby enhancing pro-inflammatory cytokine production in the inflammatory microenvironment (Gong et al., 2024). In conclusion, UAP1 plays a pivotal role in cardiovascular diseases such as AS; its interactions with lactate provide new insights into cellular functions and metabolic regulation. Ongoing research aims to further elucidate UAP1 mechanisms and explore its potential therapeutic value.

NRP1 (Neuropilin-1) is a multifunctional transmembrane protein crucial for various physiological and pathological processes, including neural development, angiogenesis, tumor progression, and immune regulation. Serving as a co-receptor for VEGF, NRP1 enhances migration, proliferation, and the formation of new blood vessels by binding to VEGF165, a process particularly vital in tumor vascularization (Gelfand et al., 2014; Hu and Jiang, 2016). NRP1 is commonly overexpressed in several cancers such as breast, lung, and pancreatic cancers, where it promotes angiogenesis to support tumor growth and increases tumor cell invasiveness and anti-apoptotic capabilities through interactions with TGF-β and integrins (Yousefi et al., 2021). Moreover, NRP1 plays a significant role in immune regulation, particularly within Tregs, boosting their interaction with dendritic cells to maintain an immunosuppressive environment and inhibit the activation of effector T cells, thus facilitating tumor immune evasion (Jung et al., 2020). In cardiovascular diseases, NRP1 aggravates the vulnerability of neovessels within atherosclerotic plaques by encouraging abnormal angiogenesis, thereby raising the risk of plaque rupture and hemorrhage. Additionally, NRP1 contributes to the formation and progression of plaques by regulating endothelial barrier functions, promoting lipid deposition, and enabling the infiltration of inflammatory cells. Its influence on inflammation operates through modulation of the TGF-β signaling pathway, enhancing macrophage activity, which in turn escalates local inflammation and leads to plaque instability (Mahdinia et al., 2023). Concurrently, the regulatory effect of NRP1 on Tregs may diminish immune clearance responses in AS, further exacerbating plaque growth and instability (Spitz et al., 2016). Lactylation, a novel post-translational modification, can directly regulate NRP1 activity and its binding affinity with ligands, impacting crucial biological functions such as angiogenesis and cell migration. Within the tumor microenvironment, lactate accumulation not only facilitates angiogenesis but also amplifies NRP1’s role in Tregs, dampening the immune response and thereby promoting tumor immune evasion (Xin et al., 2022; Wang G. et al., 2024). Future research should aim to elucidate the molecular mechanisms of NRP1 and its role in AS, especially its intricate interactions with lactate metabolism. These investigations are poised to open new avenues for developing targeted therapeutic strategies focusing on NRP1 or the lactate signaling pathway, potentially enhancing disease management by curtailing abnormal angiogenesis in atherosclerotic lesions, diminishing inflammatory responses, and maintaining immune equilibrium.

QPRT (Quinolinate Phosphoribosyltransferase), a key enzyme in the NAD^+^ biosynthesis pathway, catalyzes the conversion of quinolinic acid (QA) to nicotinamide mononucleotide (NAMN), playing a pivotal role in cellular energy metabolism, neuroprotection, and cardiovascular health across various physiological and pathological states (Youn et al., 2016; Helman and Braidy, 2023). NAD^+^ participates in cellular redox reactions, DNA repair, and inflammation regulation, with its levels also directly influencing lactate production, the development of AS, and neurodegenerative diseases. QPRT guards the nervous system by controlling quinolinic acid levels, preventing its excessive accumulation which can result in neurotoxicity and enhanced inflammation, particularly crucial in conditions such as Alzheimer’s, Parkinson’s, and Huntington’s diseases. A reduction in QPRT activity is linked to elevated quinolinic acid levels, exacerbating neurological damage and potentially speeding up the progression of AS by promoting chronic inflammation and oxidative stress (Ting et al., 2009; Sahm et al., 2013; Yan et al., 2015). Furthermore, the heightened demand for QPRT in the tumor microenvironment relates to metabolic reprogramming, especially under conditions of high glycolysis and hypoxia, where NAD^+^ regeneration is essential for maintaining lactate production and regulating lactylation modifications. Lysine lactylation, an emerging post-translational modification, impacts gene expression and metabolic regulation. Through its role in NAD^+^ metabolism, QPRT may indirectly influence lactylation modifications, affecting tumor progression and immune responses (Wang et al., 2023; Lu et al., 2024). Therefore, QPRT is crucial not only in managing neuroprotection and cardiovascular diseases but also in regulating lactate production and lysine lactylation modifications via its control of NAD^+^ levels.

NDST1 (N-Deacetylase/N-Sulfotransferase 1) is a multifunctional enzyme crucial in the biosynthesis of heparan sulfate and heparin through N-deacetylation and N-sulfation modifications. It plays a pivotal role in regulating the composition of the extracellular matrix, cell adhesion, signal transduction, and cell-matrix interactions, influencing various physiological processes (Missaghian et al., 2022). NDST1 is essential for neural development, angiogenesis, and organ morphogenesis. Abnormal expression of NDST1 is closely linked to the progression of cancer, cardiovascular diseases, metabolic disorders, and neurodegenerative diseases. In cancer, increased NDST1 activity enhances the binding of heparan sulfate to growth factors, facilitating tumor angiogenesis and metastasis (Nagarajan et al., 2018). In AS and cardiovascular diseases, NDST1’s modulation of heparan sulfate sulfation impacts endothelial homeostasis, lipid metabolism, and the proliferation of vascular smooth muscle cells. Deficiencies in NDST1 may lead to coagulation disorders, exacerbated inflammatory responses, and plaque formation (Adhikari et al., 2014; Chen et al., 2018). Additionally, NDST1 is potentially connected to lactate metabolism and lactylation modifications, which could regulate NDST1 domains, altering its activity and thereby affecting glycosaminoglycan synthesis. Particularly in tumor microenvironments and high metabolic states, lactate accumulation amplifies NDST1-mediated sulfation, increasing tumor cell proliferation and invasiveness. NDST1’s involvement in feedback regulation of cellular metabolic reprogramming, especially its interactions with lactate metabolism, may significantly contribute to the pathology of metabolic disorders and tumor progression (Gong et al., 2024; Wang G. et al., 2024; Zhou et al., 2024). Future research should delve deeper into the regulatory mechanisms of NDST1, its metabolic adaptations, and its roles in post-translational modifications to uncover new therapeutic targets for cancer, cardiovascular diseases, and other conditions.

Notably, lactate-related biology may differ across endothelial cells, macrophages, and smooth muscle cells rather than acting as a uniform plaque-wide programme. Endothelial cells are known to depend heavily on glycolysis, and recent evidence further indicates that endothelial lactate uptake and glycolytic activation can promote inflammatory activation and plaque progression in atherosclerosis (Perrotta et al., 2022; Li et al., 2025). In the present dataset, NRP1, NDST1 and UAP1 showed appreciable endothelial expression, supporting the possibility that a subset of our hub genes participates in lactate-sensitive endothelial responses related to angiogenesis, barrier dysfunction, or vascular inflammation. By contrast, macrophages likely represent the immune compartment most directly linked to lactylation-dependent transcriptional reprogramming. Histone lactylation can couple intracellular lactate accumulation to macrophage gene expression, while extracellular lactate can also modulate macrophage inflammatory signalling through GPR81, YAP and NF-κB (Zhang et al., 2019; Yang et al., 2020). Consistent with this framework, all four hub genes showed notable expression in macrophages in our single-cell data, suggesting that they may be involved in lactate-sensitive inflammatory or reparative programmes. Smooth muscle cells appear to represent a third, distinct compartment in which lactate-related metabolic remodelling may intersect with phenotypic switching. Prior work has shown that lactate can drive smooth muscle cells towards a synthetic phenotype, and metabolic reprogramming of plaque SMCs is increasingly recognised as a determinant of plaque heterogeneity and instability (Yang et al., 2017). In our study, the strong expression of UAP1, NRP1, QPRT and NDST1 in smooth muscle cells supports the possibility that these genes participate in matrix remodelling, transdifferentiation, or stress-adaptive responses within a lactate-rich plaque microenvironment.

Rivaroxaban, a direct factor Xa inhibitor, is primarily used for the prevention and treatment of venous thromboembolism and for stroke prevention in non-valvular atrial fibrillation (Carter and Plosker, 2013). Recent studies have also demonstrated Rivaroxaban’s potential therapeutic effects in AS-related diseases. By inhibiting factor Xa, Rivaroxaban effectively reduces thrombus formation and lowers the risk of thrombotic complications following the rupture of atherosclerotic plaques. Additionally, Rivaroxaban mitigates endothelial inflammatory responses and enhances vascular function by suppressing the activity of inflammatory mediators such as NF-κB, which may consequently inhibit the progression and instability of plaques (Sohma et al., 2023; Groh et al., 2024). As discussed previously, NRP1 exacerbates the instability and accelerated formation of plaques in AS by promoting angiogenesis and enhancing inflammatory responses, making it a significant therapeutic target. In our molecular docking and MD simulation studies, Rivaroxaban was found to interact with the VEGF binding site of NRP1, thereby inhibiting its activity. Through this interaction, Rivaroxaban potentially blocks NRP1-mediated angiogenesis and inflammatory responses, further decelerating the formation and progression of atherosclerotic plaques. These findings suggest that Rivaroxaban could exert a synergistic effect in treating AS by simultaneously targeting factor Xa and NRP1, providing a novel strategy for managing this condition.

In this study, we explored the relationship between atherosclerosis and lactylation-related genes by integrating multi-omics data with machine learning diagnostic models. We first analyzed public transcriptomic data from patients with atherosclerosis and control subjects to identify lactylation-related differentially expressed genes, screen hub genes, and evaluate their diagnostic value. We also investigated the immune microenvironment and cellular heterogeneity in patient-derived samples through immune infiltration analysis and single-cell RNA-sequencing analysis. We conducted a proteomics analysis on HUVECs treated with Ox-LDL *in vitro*, which revealed potential mechanisms of lactylation modification in the progression of atherosclerosis. By identifying and analyzing lactylation-modified proteins in these cells, we identified key protein changes associated with atherosclerosis, providing valuable insights for further research on the impact of lactylation modification on endothelial cell function. Furthermore, we performed additional validation in animal models using RT-qPCR, WB, and immunofluorescence staining to confirm the expression and localization of these genes *in vivo*, which further supported our preliminary findings. Additionally, we analyzed the interactions between the proteins encoded by these key genes and potential drug molecules through molecular docking and MD simulations, with particular focus on the binding of Rivaroxaban to the NRP1 protein, providing new insights and potential targets for future drug development. Overall, this study provides a lactylation-associated molecular framework for understanding atherosclerosis and offers a basis for identifying candidate biomarkers and potential therapeutic targets. Through experimental validation in both cell and animal models, together with molecular docking simulations, our findings provide supportive evidence for future mechanistic and translational studies in AS.

However, this study has several limitations. First, the 29 lactylation-associated genes used in this study were curated from previous literature and were intended to represent genes associated with lactylation biology, rather than a uniformly validated set of direct lactylation targets. Therefore, this predefined panel may introduce literature-based bias and may not fully capture novel or context-specific lactylation-associated genes in atherosclerosis. Secondly, although this study was conducted within a lactylation-associated analytical framework, we did not directly evaluate histone or protein lactylation. As a result, the present findings should be interpreted as indirect evidence linking lactylation-associated molecular signatures to atherosclerosis, rather than definitive proof of lactylation-mediated mechanisms. Thirdly, although UAP1, NRP1, QPRT, and NDST1 were prioritised through integrated multi-omics analysis and machine-learning models, their precise mechanistic relationship with lactate or lactylation remains unclear. At present, it is uncertain whether these genes are directly regulated by lactylation, indirectly influenced by broader metabolic remodelling, or simply associated with lactate-related pathological states. In addition, although key WGCNA parameters have now been described more explicitly, the identification of disease-associated modules may still be influenced by parameter selection, and further validation in independent datasets would strengthen the robustness of these findings. Moreover, while our single-cell RNA-seq analysis suggested notable expression of the identified hub genes in macrophages and smooth muscle cells, the potential differences in lactate-related metabolic or regulatory processes among endothelial cells, macrophages, and smooth muscle cells require further integrated analysis and direct experimental investigation. Finally, although molecular docking and molecular dynamics simulations suggested a possible interaction between Rivaroxaban and NRP1, this result remains computational and hypothesis-generating in nature and should not be interpreted as evidence of therapeutic repurposing without direct experimental validation. Future studies should therefore incorporate larger independent clinical cohorts, direct lactylation profiling, site-specific validation, functional perturbation experiments, and direct binding assays such as surface plasmon resonance or isothermal titration calorimetry to further clarify the biological and clinical relevance of these findings.

## 5 Conclusion

Our study identifies UAP1, NRP1, QPRT, and NDST1 as hub genes associated with lactylation-related molecular programs in atherosclerosis. These genes may serve as candidate biomarkers and hypothesis-generating targets for future mechanistic studies, but their direct regulation by lactate or lactylation remains to be established. Furthermore, single-cell sequencing analysis revealed notable expression of these genes in macrophages and smooth muscle cells involved in AS, supporting their potential roles in intercellular communication and signalling. These findings provide further insight into their possible involvement in disease progression. In addition, our research identified potential small-molecule compounds for AS treatment and explored novel mechanisms of Rivaroxaban in this disease.

## Data Availability

The datasets presented in this study can be found in online repositories. The names of the repository/repositories and accession number(s) can be found below: https://figshare.com/, doi.org/10.6084/m9.figshare.27313545.v1.
