# Supplementary figures and images for: Decoding atherosclerosis through lactylation: multi-omics integration with experimental validation
Source: Front Cell Dev Biol. 2026 May 8;14:1742425. doi: 10.3389/fcell.2026.1742425 (PMC13194442; doi:10.3389/fcell.2026.1742425)

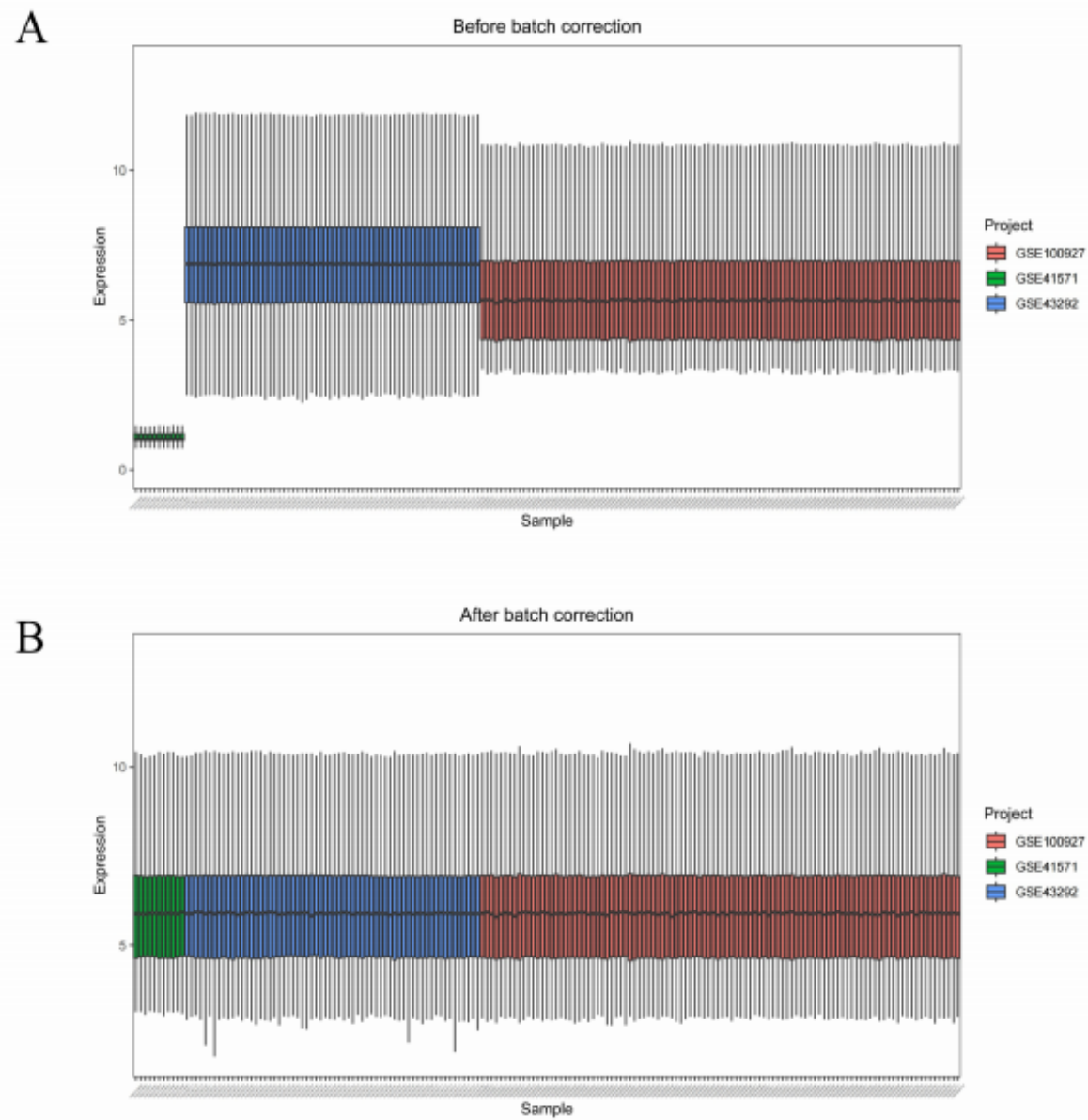

Supplementary File 5: **(A, B)** Boxplot before and after merging the three datasets: GSE100927, GSE43292, GSE41571.

Supplement: Supplementary file 1 [file Supplementaryfile5.pdf]

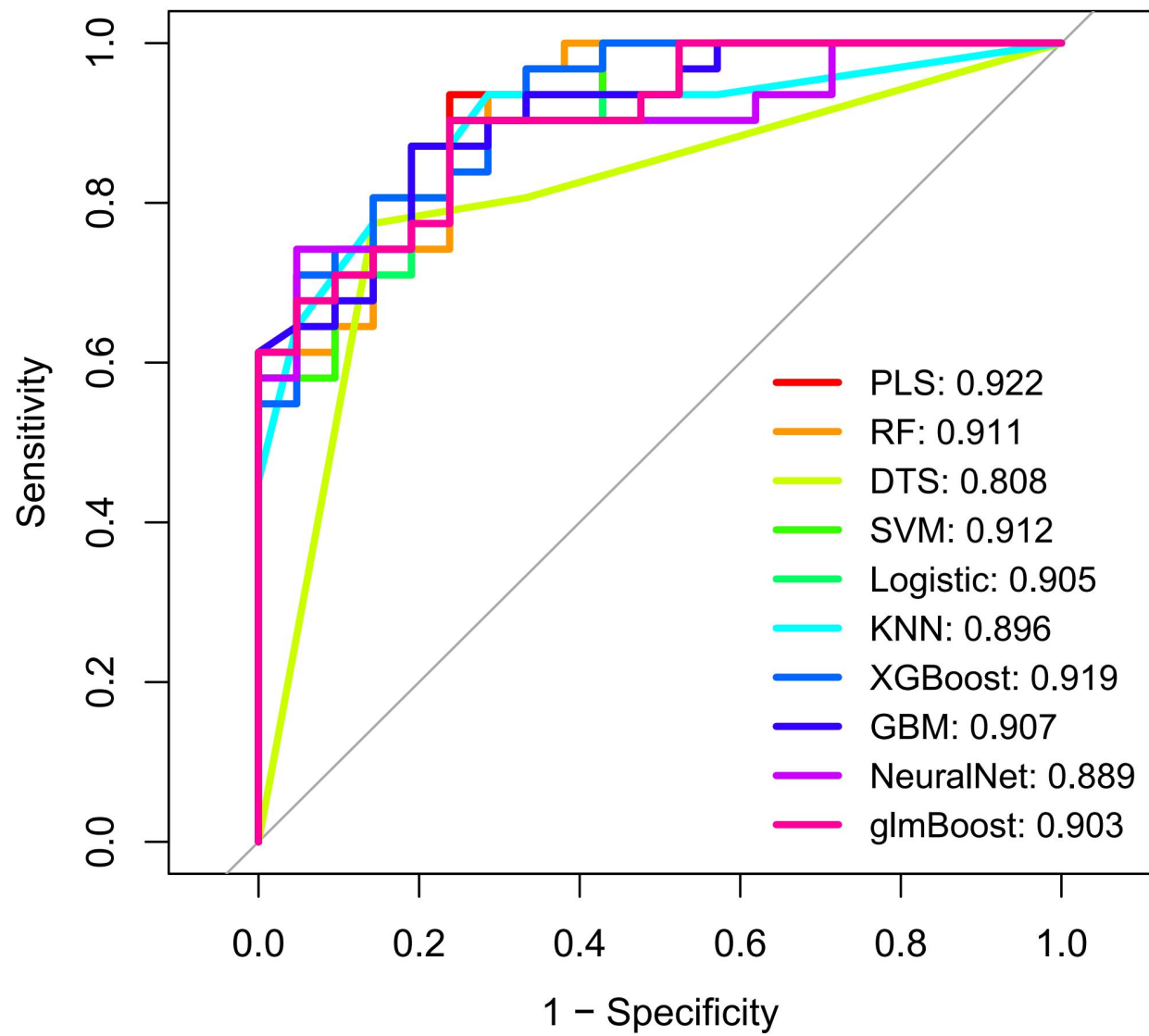

Supplementary File 6: 10 types of machine learning ROC curve plots.

Supplement: Supplementary file 8 [file Supplementaryfile6.pdf]
