# Supplementary material for: Decoding atherosclerosis through lactylation: multi-omics integration with experimental validation
Source: Front Cell Dev Biol. 2026 May 8;14:1742425. doi: 10.3389/fcell.2026.1742425 (PMC13194442; doi:10.3389/fcell.2026.1742425)
Supplement: Supplementary file 3 [file Supplementaryfile7.pdf]

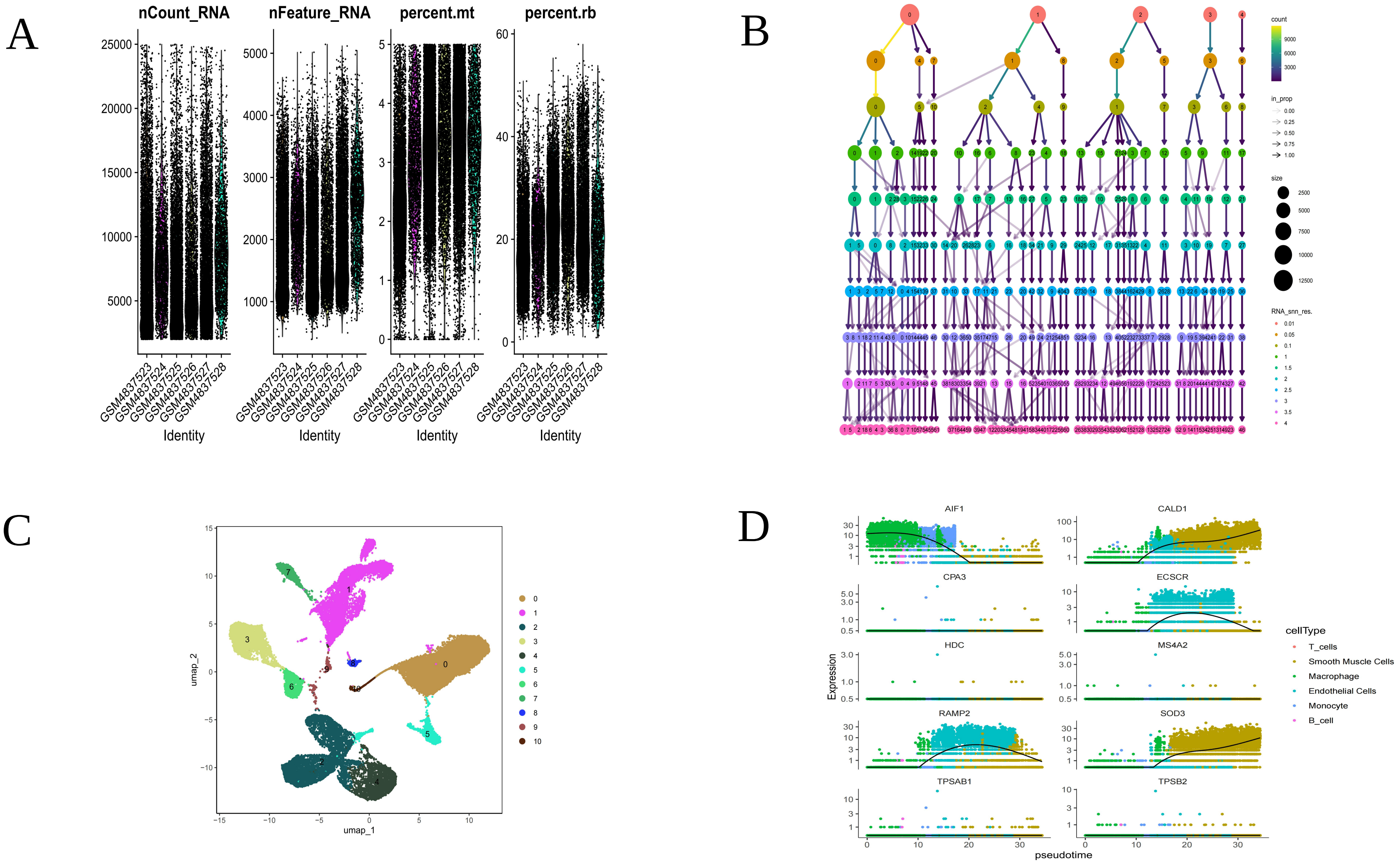

Supplementary File 7: (A) Violin plot displays single-cell RNA sequencing data for six sample groups, including the number of RNA features (nFeature_RNA), RNA counts (nCount_RNA), and the percentage of mitochondrial genes (percent.mt). (B) Cell clustering and hierarchical relationships based on single-cell RNA sequencing data, with node size and color representing the number of cells in each cluster and RNA_snn_res values, respectively. (C) UMAP visualization of cell clusters displaying the distribution of 11 distinct cell populations, each identified by a unique color. (D) Expression patterns of several genes across different cell types as a function of pseudotime, with data points representing individual cells and smoothed lines indicating average trends.
