# Supplementary material for: Decoding atherosclerosis through lactylation: multi-omics integration with experimental validation
Source: Front Cell Dev Biol. 2026 May 8;14:1742425. doi: 10.3389/fcell.2026.1742425 (PMC13194442; doi:10.3389/fcell.2026.1742425)
Supplement: Supplementary file 4 [file Supplementaryfile8.pdf]

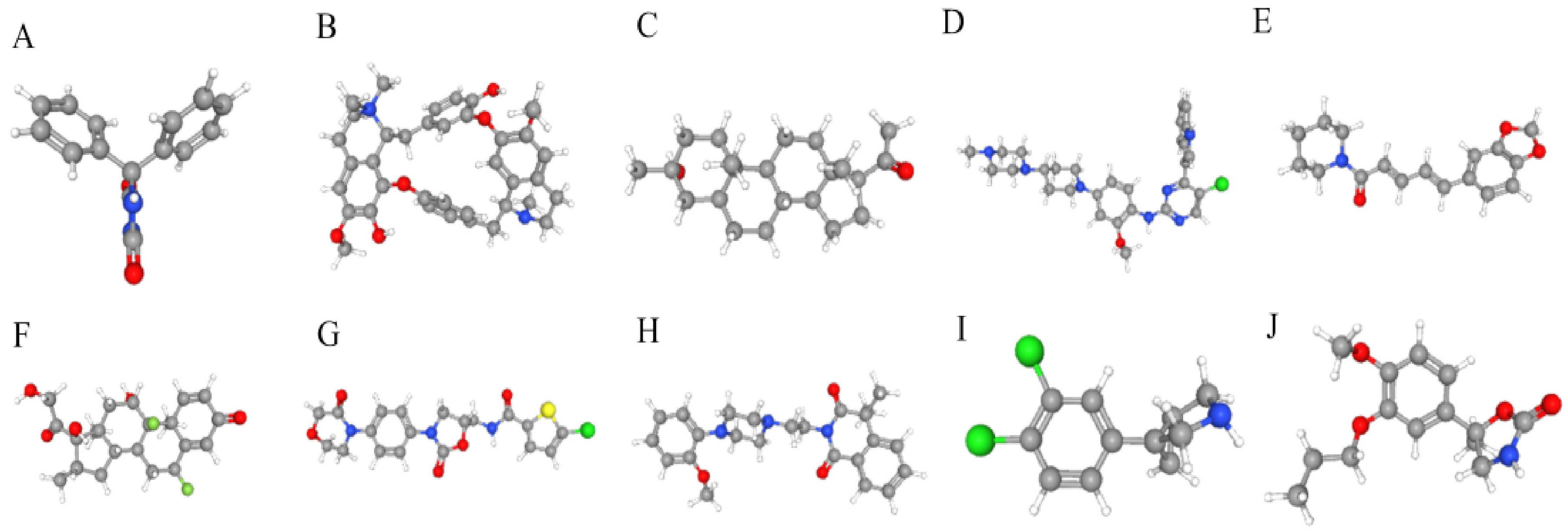

Supplementary File 8: The three-dimensional structures of phenytoin, tubocurarine, ganaxolone, HG-14-10-04, piperine, diflorasone, rivaroxaban, ARC-239, amitifadine and mesopram. (A) Phenytoin. (B) Tubocurarine. (C) Ganaxolone. (D) HG-14-10-04. (E) Piperine. (F) Diflorasone. (G) Rivaroxaban. (H) ARC-239. (I) Amitifadine. (J) Mesopram. Carbon atoms are shown in grey, hydrogen in white or light grey, oxygen in red, nitrogen in blue , sulfur in yellow, and halogen atoms in green.
