# Supplementary material for: Decoding atherosclerosis through lactylation: multi-omics integration with experimental validation
Source: Front Cell Dev Biol. 2026 May 8;14:1742425. doi: 10.3389/fcell.2026.1742425 (PMC13194442; doi:10.3389/fcell.2026.1742425)
Supplement: Supplementary file 5 [file Supplementaryfile2.docx]

Primer sequences used for RT-qPCR

| **Primer Name** | Forward (5′–3′) | Reverse (5′–3′) |
| --- | --- | --- |
| **β-actin** | ctacctcatgaagatcctgacc | cacagcttctctttgatgtcac |
| **NDST1** | gccctaaagtacacctttcatg | cgttcaatatgagtggcatacc |
| **NRP1** | agtgagaagtgtgaatggctaa | ccttcattctccccatcgatta |
| **QPRT** | catcttcactcaactcaactgc | tgttaagagccacccgttc |
| **UAP1** | agatggacggctgctgttcaatg | acatggtgctgcaactgaggttc |
