# Supplementary material for: Decoding atherosclerosis through lactylation: multi-omics integration with experimental validation
Source: Front Cell Dev Biol. 2026 May 8;14:1742425. doi: 10.3389/fcell.2026.1742425 (PMC13194442; doi:10.3389/fcell.2026.1742425)
Supplement: Supplementary file 6 [file Supplementaryfile4.pdf]

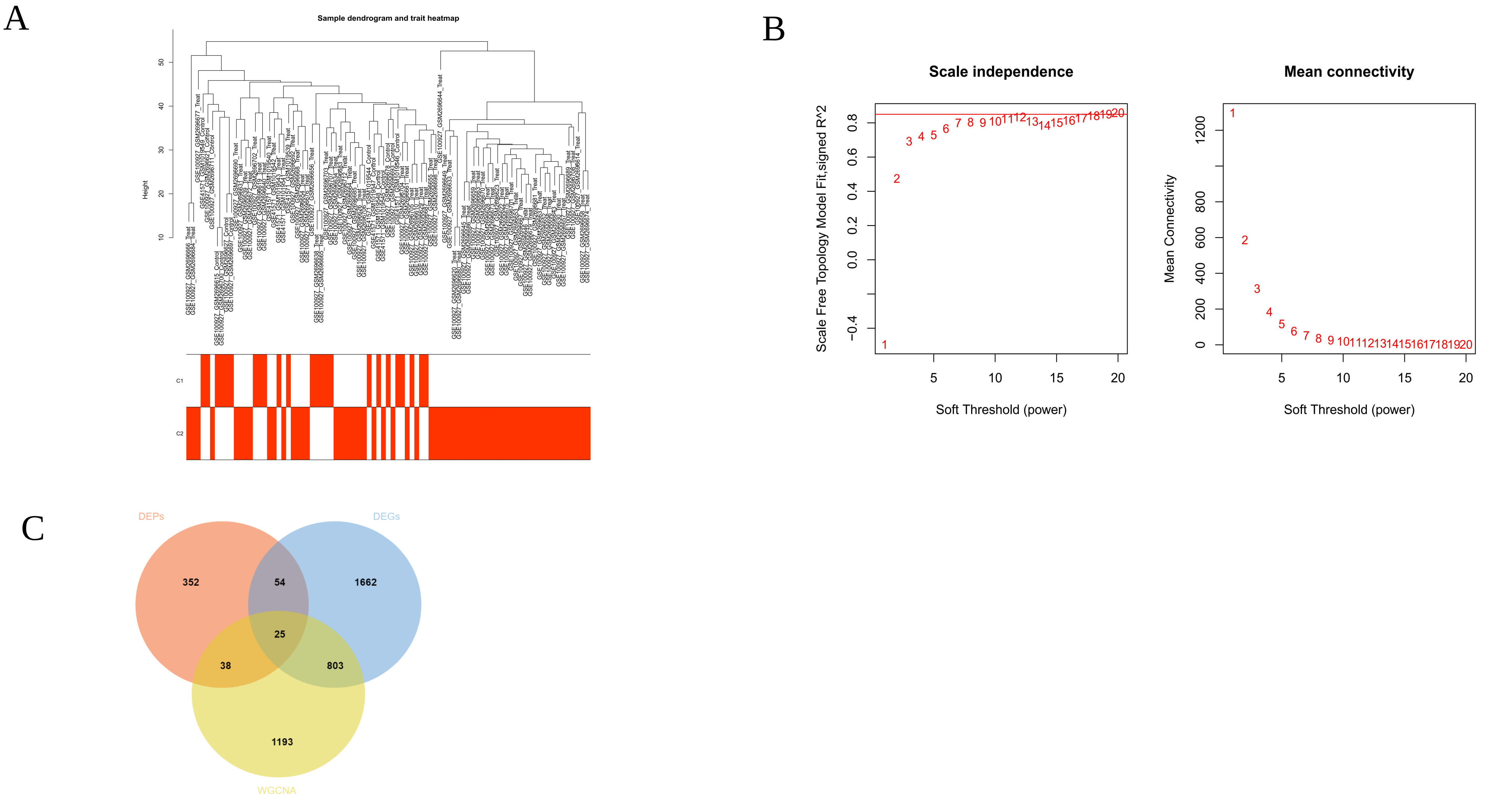

Supplementary File 4: (A) Sample dendrogram and trait heatmap illustrating the distribution of traits across the main categories C1 and C2. (B) The determination of soft thresholding power. (C) Venn diagram depicting common genes between DEPs, DEGs and key module genes.
