# Supplementary material for: Decoding atherosclerosis through lactylation: multi-omics integration with experimental validation
Source: Front Cell Dev Biol. 2026 May 8;14:1742425. doi: 10.3389/fcell.2026.1742425 (PMC13194442; doi:10.3389/fcell.2026.1742425)
Supplement: Supplementary file 7 [file Supplementaryfile3.pdf]

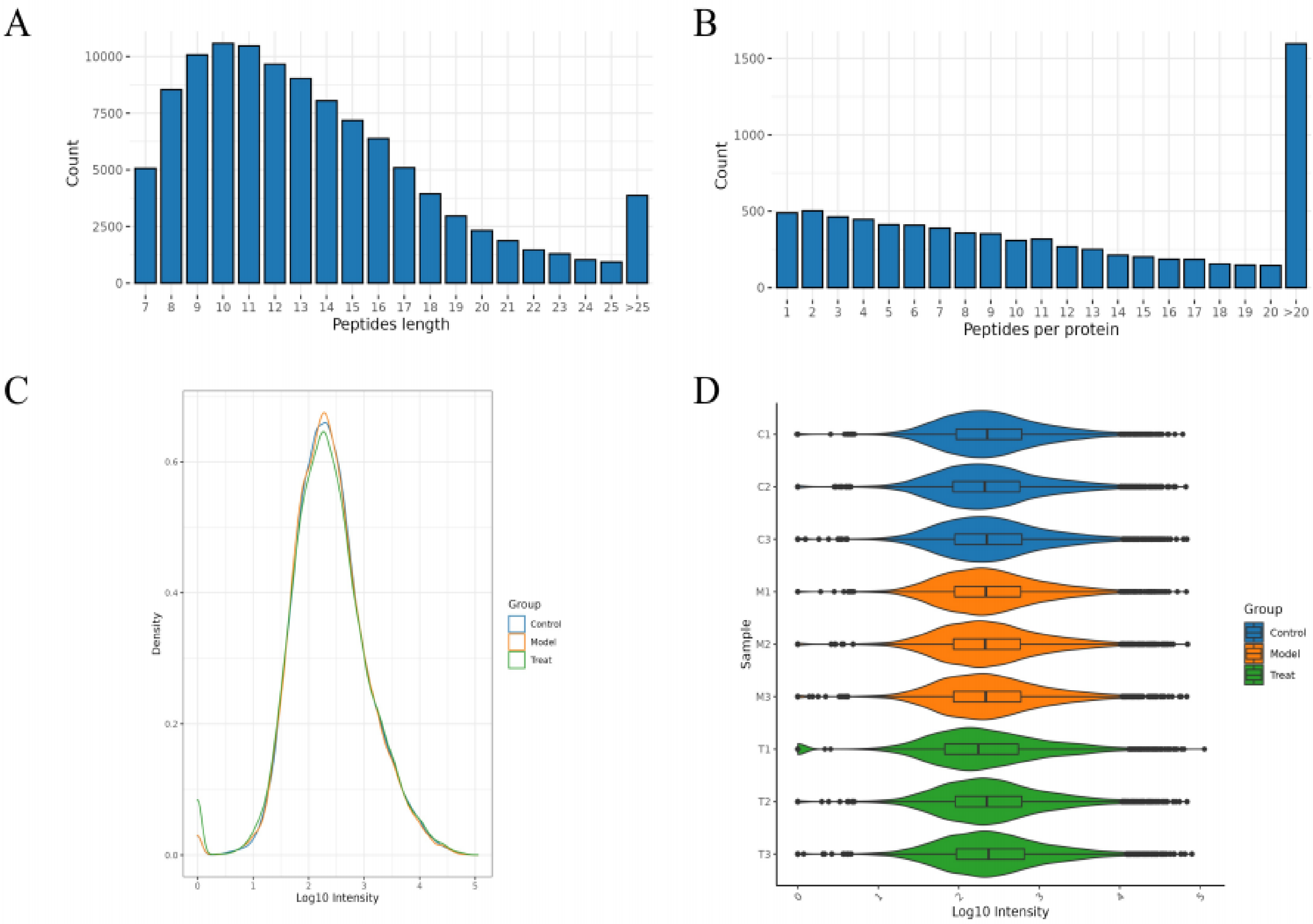

Supplementary File 3: Proteomics data. **(A, B)** Bar plots show the distribution of peptide lengths and the distribution of peptides per protein. **(C)** Density plot shows the distribution of Log10 intensity values across the groups. **(D)** Violin plot shows the distribution of Log10 intensity values for samples.
